# Machine learning for the meta-analyses of microbial pathogens’ volatile signatures

**DOI:** 10.1038/s41598-018-21544-1

**Published:** 2018-02-20

**Authors:** Susana I. C. J. Palma, Ana P. Traguedo, Ana R. Porteira, Maria J. Frias, Hugo Gamboa, Ana C. A. Roque

**Affiliations:** 1 UCIBIO, REQUIMTE, Departamento de Química, Faculdade de Ciências e Tecnologia, Universidade Nova de Lisboa, 2829-516 Caparica, Portugal; 2 LIBPhys-UNL, Departamento de Física, Faculdade de Ciências e Tecnologia, Universidade Nova de Lisboa, 2829-516 Caparica, Portugal

## Abstract

Non-invasive and fast diagnostic tools based on volatolomics hold great promise in the control of infectious diseases. However, the tools to identify microbial volatile organic compounds (VOCs) discriminating between human pathogens are still missing. Artificial intelligence is increasingly recognised as an essential tool in health sciences. Machine learning algorithms based in support vector machines and features selection tools were here applied to find sets of microbial VOCs with pathogen-discrimination power. Studies reporting VOCs emitted by human microbial pathogens published between 1977 and 2016 were used as source data. A set of 18 VOCs is sufficient to predict the identity of 11 microbial pathogens with high accuracy (77%), and precision (62–100%). There is one set of VOCs associated with each of the 11 pathogens which can predict the presence of that pathogen in a sample with high accuracy and precision (86–90%). The implemented pathogen classification methodology supports future database updates to include new pathogen-VOC data, which will enrich the classifiers. The sets of VOCs identified potentiate the improvement of the selectivity of non-invasive infection diagnostics using artificial olfaction devices.

## Introduction

Infectious diseases represent an enormous human and economic burden to modern societies. Unfortunately, such weight is expected to increase with the rise of antimicrobial resistant pathogens, already considered worldwide an alarming situation in public health. The early identification of the infectious agent allows the prompt initiation of appropriate antimicrobial therapy, reducing healthcare costs and patient discomfort, while also contributing to refrain the spreading of antimicrobial resistant pathogens^1^. The traditional methods for microorganism identification rely on culture of clinical samples, which might take up to a week to retrieve results. These methods are often complemented by more precise molecular diagnostics techniques, which detect known biomarkers - e.g. bacterial cell-surface antigens or bacterial-specific nucleic acid sequences – for the identification of infectious agents. Still, these are also invasive, time-consuming and expensive methods. The search for fast infection diagnostic tools is therefore a priority, and non-invasive diagnostic devices, in particular those exploring the volatolomics^2^ concept, have the potential to contribute to this challenge^3,4^.

The human body produces a diversity of organic compounds as a result of its normal metabolism. Many of these compounds are volatile: lipophilic small molecules with high vapour pressures and low boiling points that can easily evaporate, being released into different body fluids as blood, breath or faeces^5,6^. The production of new volatile organic compounds (VOCs), or the alteration of the normal pool of VOCs, has been associated with several diseases^3^ including cancer^7,8^, pneumonia^9^, tuberculosis^10^, and coeliac disease^11^. Pathogenic microorganisms such as bacteria and fungi also release a variety of VOCs to the environment. Microbial VOCs are involved in functions such as intra- and inter-species communication, growth regulation, pathogenicity and stress resistance^12,13^. Combinations of VOCs representing pathogen signatures, could thus be explored for diagnosis of infectious diseases. In this context, electronic noses have been successfully used for the discrimination of certain pathogens, processing signal patterns generated in the presence of different microbial species, although without acknowledging the exact nature of the VOCs present in the samples^4,14,15^. VOC-selective gas sensing devices have the potential to reduce the complexity of electronic noses’ sensing arrays and signal processing load. However, the identification of VOC signatures associated with microbial pathogens is still inexistent, clearly representing a major obstacle towards selective gas-sensing diagnostics.

The search for microbial VOCs as infection biomarkers has intrigued several scientists in the past, who made use of sensitive analytical laboratorial equipment, as gas chromatography coupled to mass spectrometry (GC-MS) or selected-ion flow-tube mass spectrometry (SIFT-MS) (detection limits in the range of ppt_v_-ppb_v_), to analyse the headspace of microbial cultures or patient samples^16–24^. The use of distinct sample sources, testing conditions, sampling methods and analytical techniques contributes to the large amount of available data scattered in the bibliography, making data interpretation a challenging task. Previous review works compiled information published up to 2016^25–29^ and compared lists of VOCs emitted by different microorganisms. For most species, there is not an accepted univocal VOC-microorganism association for the identification of the infection agent in biological samples.

Machine learning deals with large and diverse datasets to extract relevant information, being an increasingly critical computing tool in ecology^30^, healthcare and life sciences^31–33^. Artificial intelligence is also considered important for the control of infectious diseases^34,35^. Unsupervised machine learning methods have been used to determine that the pathogenicity and non-pathogenicity of microorganisms is associated with similar combinations of emitted VOCs^36^. However, the discrimination of human pathogen species by VOC patterns has never been approached with supervised machine learning methods using published data. The current work aimed at filing this gap. A wide and rich dataset correlating released VOCs (from *in vitro* culture headspaces or clinical samples) with microbial agents was generated, by assembling the reports published between 1977 and 2016. This databank has the potential to be expanded in the future as new reports become available. Machine learning methods based on support vector machines (SVM)^37^ and features selection were then applied to identify subsets of microbial VOCs that contribute for the accurate distinction between several microbial pathogens relevant in clinical settings. Such unique information provides the basis to bring gas-sensing diagnostics to the level of clinical acceptance of molecular diagnostics, as microbial VOCs contribute to the sensitive and accurate detection of infectious agents, integrated in fast, non-invasive sensing devices.

## Results

### Data collection and selection

**Figure 1:**
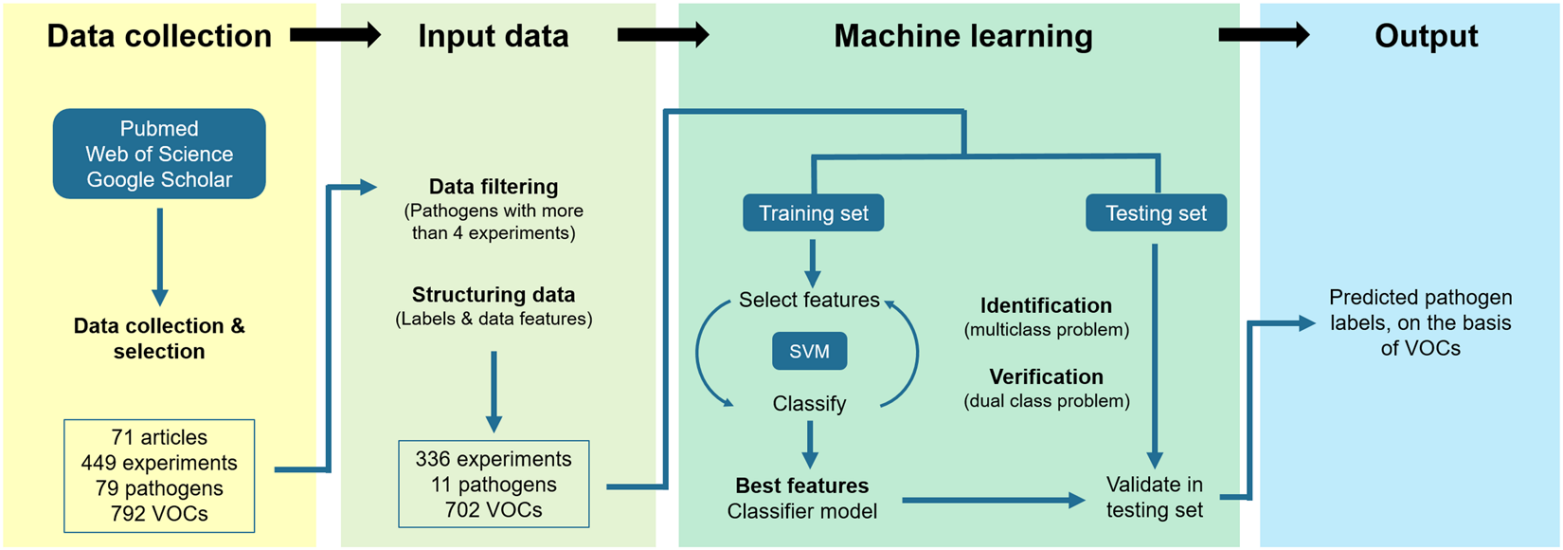
Research strategy. The workflow was divided in four main tasks, including data collection, input data, machine learning and output data. The selected data available in the literature was organized in a matrix of labels (pathogens) and features (VOCs), and further used as the input for machine learning steps. Feature selection and classification algorithms were implemented using support vector machines (SVM) to determine the set of VOCs that better separates the pathogens, and build a model that predicts the pathogen based on information about the presence/absence of a set of VOCs in a sample.

The research strategy followed in this work is schematically presented in Fig. 1, showing the four main stages (i) collection, selection and descriptive analysis of data from literature; (ii) preparation of input data in the form of a pathogen-VOC matrix; (iii) application of machine learning tools to generate a classifier model, and (iv) resulting putative microbial VOC biomarkers as output data. In the first stage, a comprehensive literature search in the online databases of scientific publications retrieved approximately 4000 articles (951 from Web of Science, 801 from PubMed and 2186 from Google Scholar) reporting microbial VOCs. After excluding reviews and conference articles, the remaining documents were screened based on content, through title, abstract and full text (Supplementary Fig. S1). Cancer and chronic respiratory disease-related publications were not considered. The number of relevant articles was thus narrowed down to 71 (Supplementary Tables S1 and S2), from which the information about VOCs emitted by microorganisms associated with human diseases was collected. The 71 articles include data from 449 experiments, involving 79 microbial pathogen species and 792 VOCs (Fig. 1). In this work, an experiment was defined as a pathogen’s VOC dataset obtained in specific experimental conditions, and it was often the case that one publication corresponds to several experiments.

The interest in studying released VOCs for the distinction of pathogens is not recent. The first relevant study was published in 1977 and applied GC-MS to detect VOCs in the headspace of *Escherichia coli* and *Proteus mirabilis* cultures^38^. There were few publications until 2005 (less than 1 per year) but from then onwards there was a strong increase in research, coherent with the evolution of the analytical methods and the interest in developing rapid techniques for infection detection. Most articles (86%) were published in the last 11 years (2005–2016), with the maximum number of studies being published in 2016 (Supplementary Fig. S2a). Different analytical methods were employed in the studies (Supplementary Fig. S2b), and in some reports the results were obtained with more than one method (for example GC-MS and IMS^39^ or GC-MS, SIFT-MS and SESI-MS^40^). The three most used methods were GC-MS, SIFT-MS and GC, accounting for 80% of the collected data, but the gold-standard for VOCs detection and quantification is GC-MS. This technique was employed in the majority (~60%) of the studies and generated a large part of the experiments (45%) used in this work. It is also interesting to note that half of the papers published in 2016 used two-dimensional GC coupled with time-of-flight mass spectrometry (GC X GC ToFMS), showing the increasing relevance of this technique, able to discriminate over a larger range of compounds due to the increase of compound resolution given by the second dimension.

To develop techniques for fast microbial detection using VOC biomarkers in clinical settings, the ideal sources of pathogen VOCs would be patient samples – body fluids or breath – especially those with already confirmed microbiological diagnosis. However, only a minority (6%) of the experiments described in the literature used clinical samples for VOC analysis (Supplementary Fig. S2c). The most used type of clinical sample is breath (that represents only 3% of the total number of experiments), probably due to the easy collection method and to the impact of respiratory diseases. An alternative approach consists in the collection of the clinical isolate (from patient samples) followed by collection of the headspace of a pure culture for identification of released VOCs. Typically, the culture medium alone is analysed in parallel with the microbiological cultures, and only the VOCs with differential expression relative to the culture medium are considered microbial VOCs^16^. Clinical isolates are the second most used samples for VOCs detection (30%), obtained from blood, respiratory fluids (e.g., sputum, tracheal aspirates), urine and skin (Supplementary Fig. S2c). Most of the papers report experiments with reference strains (60%), well-characterized commercial laboratorial strains.

**Figure 2:**
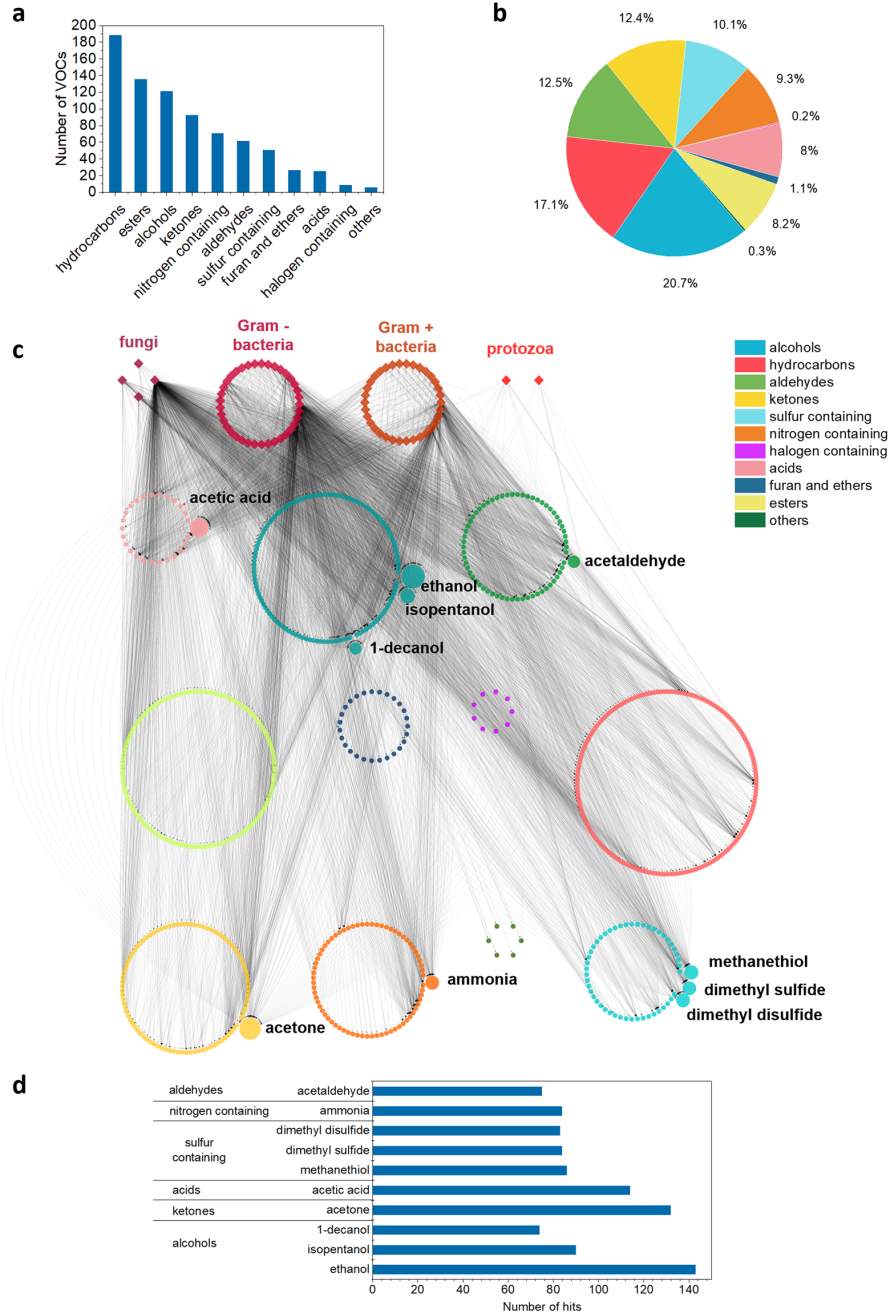
Classes and diversity of VOCs emitted by microbial pathogens. **(a)** Number of different VOCs from each chemical class. **(b)** Relative abundance of VOC chemical classes, given by the ratio between the number of hits of VOCs in a chemical class and the number of hits of VOCs in all classes. **(c)** Graphical representation of pathogen-VOC associations described in the literature (Cytoscape 3.5). Each line represents one hit for a given pathogen-VOC association; the diameters of the circles that represent each chemical class are proportional to the number of different VOCs within that class. **(d)** Number of hits and chemical class of the 10 most referred VOCs, also highlighted in **(a)**.

The 79 reported pathogens were grouped between Gram-positive bacteria (34), Gram-negative bacteria (39), *fungi* (4) and protozoa (2) (Fig. 2c, Supplementary Fig. 2d and Table S2). *Pseudomonas aeruginosa*, *Escherichia coli* and *Staphylococcus aureus*, ranked as priority warning bacteria by the World Heath Organization (WHO)^41^, are the most studied pathogens, reported in more than 20 publications and involved in more than 40 experiments each. On the opposite extreme, there are pathogens with only one report (e.g., *Acinetobacter baumannii*, *Plasmodium falciparum* or *Legionella pneumophila*) (Supplementary Fig S2d).

Raw data from the experiments listed in Supplementary Fig. S2d was collected to an extensive in-house built database. Mainly qualitative VOC data was reported in the publications, i.e., presence/absence of a VOC in a sample or increase/decrease of a VOC concentration compared to negative controls. Only 7 out of the 71 publications (91 of the 449 experiments) refer concentrations of the detected VOCs^20,42–46^. Within the scope of the present study, the available quantitative VOC data was converted into binary data (VOC is present or absent in the sample) to be included in the descriptive analysis of the state of the art and in the machine learning VOC-pathogen dataset matrix.

Among the 792 different VOCs emitted by human microbial pathogens, most of them belong to the hydrocarbon (189), ester (136), alcohol (122) and ketone (93) chemical classes. These are the classes of compounds with higher structural variability (Fig. 2a and c). On the other hand, alcohols are the most abundant compounds among the volatiles emitted by pathogens, representing 21% of all the hits, in good equilibrium with hydrocarbons (17%) and followed by aldehydes and ketones (both with 12% of the total hits) (Fig. 2b). In absolute terms, ethanol (143 hits), acetone (132 hits), acetic acid (114) and isopentanol (90 hits) are the most reported VOCs (Fig. 2d). One hit for a given VOC was defined as one observation of the VOC in one experiment reported in the literature. Therefore, the number of hits of a given VOC is the total number of experiments in which the VOC was detected.

### Machine learning

Most VOCs listed in this work are emitted by more than one pathogen (shared VOCs, e.g. ethanol), while others have been reported exclusively for one specific pathogen (exclusive VOCs, e.g. ethane, for P. aeruginosa). Besides the fact that not many pathogens have exclusive VOCs that can be used as the pathogen’s biomarker (Figure S3), most of the exclusive pathogen-VOC associations still lack confirmation as they were found in just a few reports (Figure S3 and Table S4). On the other hand, due to the complexity of pathogen-VOC associations (Fig. 2 and Supplementary Fig. S3), manually comparing VOC emission profiles to detect pathogen VOC signatures is not feasible. Machine learning algorithms can expedite the pathogen distinction task as they provide automatic methods to separate similar data, based on pattern analysis^3,31^. For example, a recent study employed unsupervised machine learning (cluster analysis) to group microorganisms according to the similarity of the emitted VOC profiles^36^. It was concluded that pathogens emit similar combinations of VOCs, which allow to distinguish them from non-pathogenic microorganisms.

We hypothesise that, beyond the VOC profiles similarity between pathogens^36^, there might be intrinsic, but not obvious, differences that carry sufficient information to distinguish pathogens at the species level. To evaluate this hypothesis, supervised machine learning tools were used.

In supervised learning, there are several input variables associated with features of the problem (in this work, the VOCs are the features, and presence or absence of those VOCs are the inputs), an output variable (in this work, the pathogen) and an algorithm (a classifier) that learns a mapping function from the input to the output, in an automatic manner. The learning is performed with training examples (those for which the input and output are known) and the goal is to learn the mapping function so well that when new input data is submitted, the algorithm can predict the output (Figure S6).

In the context of this work, the aim was to determine the VOCs with major pathogen discrimination power and devise pathogen classifiers to predict the pathogen identity when data on presence and absence of those VOCs on a sample is supplied as input.

**Figure 3:**
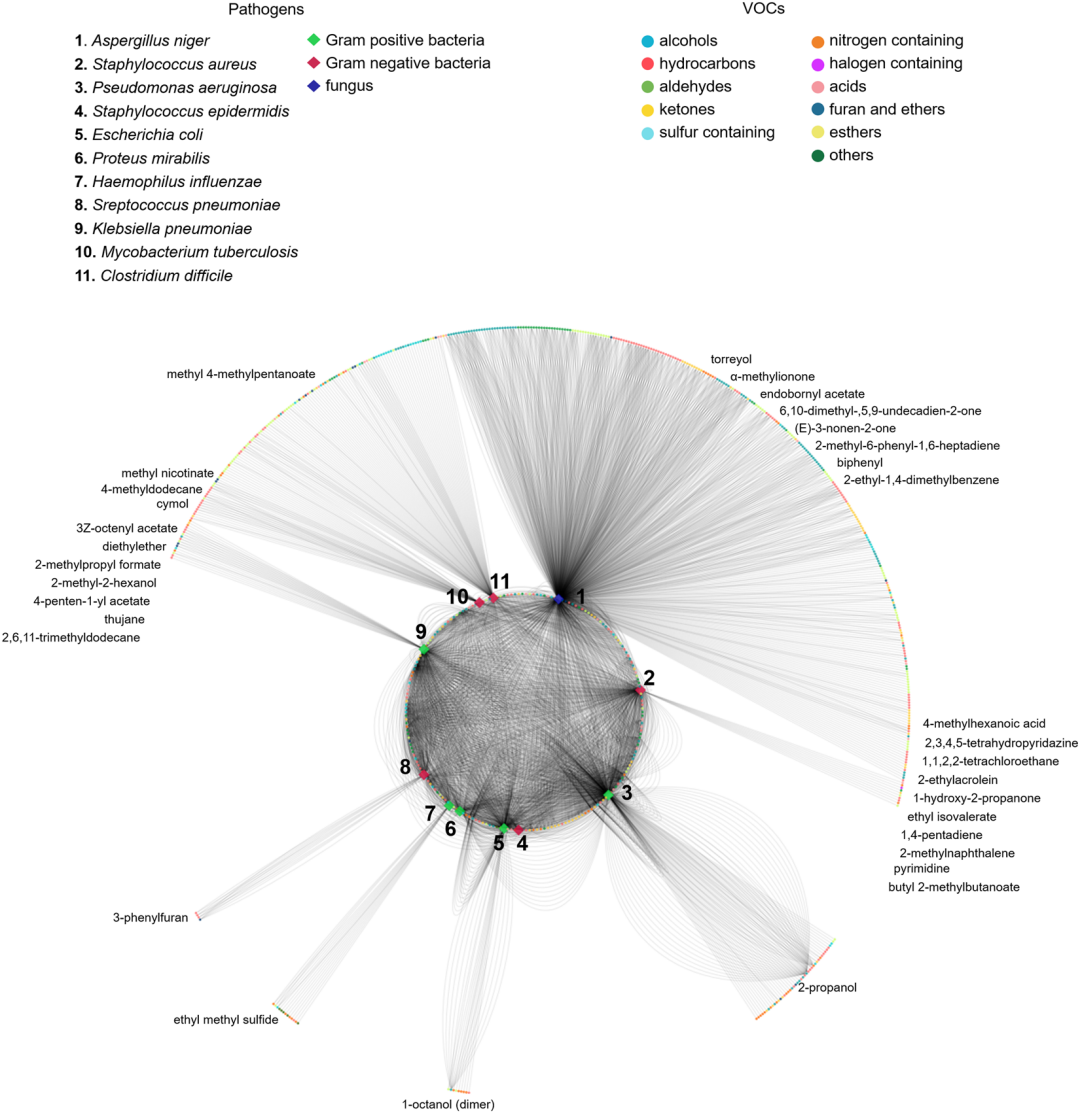
Graphical representation of the associations between the 11 pathogens with more than 4 experiments and the 702 VOCs identified in the scope of a total of 336 experiments. Each line represents one hit for a given pathogen-VOC association; VOC nodes within the circumference represent VOCs that were reported to be emitted by multiple pathogens, while VOC nodes outside the circle represent VOCs that were reported to be emitted by only one pathogen. The most referred exclusive VOCs per pathogen are indicated outside the graph.

A correct design of a classification algorithm should implement a validation mechanism with sufficient examples per class to be divided in training and testing datasets. However, the number of examples available for each the 79 pathogens is unbalanced (Supplementary Fig. S2d). For example, while there are 84 experiments with VOC data for *Escherichia coli*, there is only one for *Legionella pneumophila*. This reflects the research efforts dedicated to the different pathogens, but limits the amount of usable data for the adequate implementation of automated pathogen classifiers. The pathogen-VOC database was, thus, filtered to include only the pathogens for which more than 4 experiments have been reported (Fig. 3). It was also reorganized in the form of a matrix where each line represents an example (an experiment) for a pathogen species and each column represents a VOC (presence/absence of one VOC) (Supplementary Table S9).

### Input data for machine learning

The filtered dataset consists of a 336 × 702 binary matrix, corresponding to the associations between the 11 pathogens with more than 4 experiments and the 702 VOCs identified in the scope of a total of 336 experiments.

Top hit VOCs for each pathogen were found (Supplementary Table S3), as well as an associated set of exclusive VOCs (Fig. 3 and Supplementary Table S4) for all pathogens except *Staphylococcus epidermidis* and *Proteus mirabilis*. The only pathogens for which the detection of the same exclusive VOCs was repeatedly reported in different publications are *Mycobacterium tuberculosis*, with cymol^10,47,48^, methyl nicotinate^47,49,50^ and 4-methyldodecane^48,51,52^ and *Pseudomonas aeruginosa*, with 2-propanol (although reported in 37 experiments, 36 of them were from the same source^43,53^).

Another interesting perspective is to identify compounds shared by all pathogens, as they could be employed as putative infection indicators (or non-infection indicators, if absent). Among the total 702 VOCs, acetaldehyde is the only common to all 11 pathogens (Supplementary Table S5). It has been identified in the headspaces of reference strains cultures (12 ppbv – 11 ppmv)^20,42,45,46^ in the headspaces of clinical isolates from respiratory fluid (ppmv - pptv) with *Streptococcus pneumoniae and Haemophilus influenzae*^54^, and in headspaces of clinical isolates from blood infected with *Klebsiella pneumoniae*, *Escherichia coli, Proteus mirabilis, Pseudomonas aeruginosa, Staphylococcus aureus, Staphylococcus epidemidis and Streptococcus pneumoniae*^51,55^. It was also detected in exhaled breath of *Mycobacterium tuberculosis*^51^ and *Pseudomonas aeruginosa* infected patients (8 ppbv median concentration)^44^ and in the headspace of faeces of patients infected with *Clostridium difficile*^56^. Despite being a ubiquitous compound in body fluids and breath of healthy subjects^5^; the information about its physiological range of concentrations is scattered. In exhaled air, the detection of acetaldehyde has been associated with oral cancer, chronic alcohol consumption and smoking^57^ but may also be related with oral hygiene^58^, therefore, its use as infection indicator should be considered carefully. Other VOCs were also found to be common for most of the 11 pathogens in the filtered dataset. Namely, dimethyl sulfide, dimethyl disulfide, acetic acid, 1-propanol, toluene, ethyl acetate and methanol are shared between at least 9 out of the 11 pathogens (Supplementary Table S5).

### Automatic classification of pathogens using machine learning

Two different approaches were taken for the automatic classification of pathogens based on released VOCs: (i) multiclass and (ii) dual class modes.

The multiclass mode is also called identification mode because there are several classes and the goal is to identify to which class new data belongs to. The question addressed in this mode is: “Based on these VOCs, which is the most probable pathogen present in the sample?”

From the 702 VOCs in the filtered dataset, a profile with the 18 most discriminating VOCs was selected by the SVM-based classification algorithm to separate the 11 pathogens in the multiclass mode (Table 1). In practice, this implies that to predict the identity of a pathogen with its maximal accuracy (76.5%), the classifier only needs the binary information (presence or absence in the sample) about those 18 VOCs, out of the 702 in the 11 pathogen – 702 VOC database. For example, if information (absence or presence in a sample) about VOC1 (1-decanol) is provided to the classifier, the accuracy of the classification made with only this information will be, in average, 49.7%. On the other hand, if information about all the 18 VOCs in Table 1 is supplied, the accuracy of the prediction will increase to 76.5% (in average).

**Table 1:** List of VOCs that lead to the best classification results in the identification mode of the classifier, considering the 11 pathogen – 702 VOC dataset and using “leave-one-out” cross validation. The VOCs are listed in descendent order of importance for the performance of the classifier. Classification accuracy improves gradually by the sequential addition of the VOCs in the list to the vector of features that is used to classify the samples.

Number of VOCs used	VOC	Chemical structure	Added identification accuracy (%)	Cumulative identification accuracy (%)
1	1-decanol		49.7	49.7
2	3-methylbutanal		3.3	53.0
3	ethyl acetate		3.0	56.0
4	1,3,5-trimethylbenzene		1.7	57.7
5	3-methylbutanoic acid		1.8	59.5
6	indole		2.4	61.9
7	isopentanol		3.0	64.9
8	1-undecene		2.7	67.6
9	2-methylbutanal		2.0	69.6
10	ɣ-butyrolactone		1.5	71.1
11	4-methylphenol		0.9	72.0
12	furan		1.2	73.2
13	cymol		0.9	74.1
14	methyl nicotinate		0.9	75.0
15	cyclohexanone		0.6	75.6
16	4-methylpentanoic acid		0.2	75.8
17	n-butyl acetate		0.4	76.2
18	1-butanethiol		0.3	76.5

The performance of the model was evaluated using the “leave-one-out” cross-validation method to access its ability to make predictions on unseen data. Although there is an error associated with the classification, that error is known for each pathogen, as represented in the confusion matrix (Table 2) and quantified by the sensitivity and precision of the classifier (Supplementary Table S6). For example, if the classification is “*Mycobacterium tuberculosis”*, there is 100% certainty of having it in the sample; If the classification is “*Klebsiella pneumoniae”*, there is 84% certainty in the result (Supplementary Table S6).

**Table 2:** Confusion matrix illustrating the prediction results of the classifier in the identification mode for the 11 pathogen – 702 VOC dataset. underlined bold cells represent the incorrect predictions made by the classifier and **bold** cells represent the correct predictions.

		Predicted pathogen										
		*A. niger*	*C. difficile*	*E. coli*	*H. influenzae*	*K. pneumoniae*	*M. tuberculosis*	*P. mirabilis*	*P. aeruginosa*	*S. aureus*	*S. epidermidis*	*S. pneumoniae*
**Actual pathogen**	*A. niger*	**8**	0	0	0	0	0	0	0	0	0	0
	*C. difficile*	0	**6**	0	0	0	0	0	0	0	0	0
	*E. coli*	0	0	**60**	0	**2**	0	**2**	**19**	**1**	0	0
	*H. influenzae*	0	0	**1**	**5**	0	0	0	0	**1**	0	0
	*K. pneumoniae*	0	0	0	0	**16**	0	0	**6**	**11**	0	0
	*M. tuberculosis*	0	0	0	0	0	**9**	0	0	0	0	0
	*P. mirabilis*	0	0	0	0	**1**	0	**6**	**5**	**2**	0	0
	*P. aeruginosa*	0	0	**2**	0	0	0	0	**114**	**2**	0	0
	*S. aureus*	0	0	**2**	0	0	0	0	**11**	**29**	0	0
	*S. epidermidis*	0	0	0	0	0	0	0	**1**	**1**	**3**	0
	*S. pneumoniae*	0	0	**1**	0	0	0	0	**4**	0	0	**5**

Overall, the application of the classifier in unseen data resulted in 261 true positives (correctly predicted pathogens) in a total of 336 examples, but the classification performance was better for some pathogen classes than others (Table 2 and Supplementary Table S6). Namely, the classifier is very sensitive (100%) and precise (100%) regarding *Aspergillus niger*, *Clostridium difficile* and *Mycobacterium tuberculosis*, which were never confused with other pathogens. *Pseudomonas aeruginosa*, *Escherichia coli* and *Haemophilus influenzae* were also predicted with high sensitivity (>70%) and precision (>70%). On the other hand, *Proteus mirabilis* was more misclassified than correctly predicted (43% sensitivity and 75% precision): the model confused this pathogen with *Klebsiella pneumoniae*, *Pseudomonas aeruginosa* and *Staphylococcus aureus*. The fact that *Proteus mirabilis* only emits shared VOCs (Fig. 3), and the low number of experiments available for this pathogen compared to other classes (Fig. 2d), might contribute to the low sensitivity of the model to identify *Proteus mirabilis*. A similar situation happened with *Klebsiella pneumoniae* (49% sensitivity and 84% precision), which was mislabelled 17 times: 6 times as *Pseudomonas aeruginosa* and 11 times as *Staphylococcus aureus*.

The performance of the multiclass classifier indicates that the complexity of the published pathogen-VOC associations can be reduced and that there is a minimal set of VOCs with pathogen discrimination power, sufficient to predict the identity of some pathogens.

In a different approach, pathogen classification was interpreted as a dual-class (verification) problem. Here, the goal is to verify whether a set of VOCs corresponds to a specific pathogen or to any other. This is also called verification because the classifier verifies if the assumption of the data belonging to a specific class is true. This type question is answered: “Does the given VOC data belong to, *e.g*., *Klebsiella pneumoniae*?”.

For each pathogen class, the implemented SVM classifier selected the set of VOCs that allows the best separation from all the others. High discrimination accuracies (>90%) and precisions (>86%) were achieved, while the sensitivity was variable (Table 3).

**Table 3:** Prediction results of the classifier in the verification mode, for the 11 pathogen – 702 VOC dataset and using “leave-one-out” cross-validation.

Pathogen	No. of exps.	Verification VOCs set	Base accuracy (%)	Computed accuracy (%)	Computed sensitivity (%)	Computed precision (%)
*A. niger*	8	(E)-3-nonen-2-one	97.6	100	100.0	100.0
*C. difficile*	6	methyl 4-methylpentanoate 4-methylpentanoic acid 1-methyl-2-(1-methylethyl)-benzene	98.2	100	100.0	100.0
*E. coli*	84	indole 2-pentanone 1-octanol 1-ethyl-2-methylbenzene isoamyl acetate (Z)-7-tetradecen-1-ol 1-undecene 3-methylbutanoic acid hexanal phenylmethanol	75.0	90.1	66.7	94.9
*H. influenzae*	7	ɣ-butyrolactone 1,2-bis(trimethylsilyl)benzene^(a)^	97.9	99.4	85.7	85.7
*K. pneumoniae*	33	2,2,4,4-tetramethyloxolane 3Z-octenyl acetate 3-methylcyclohexene	90.2	91.7	15.2	100.0
*M. tuberculosis*	9	4-methyldodecane cymol methyl nicotinate	97.3	100	100.0	100.0
*P. mirabilis*	14	(1,1-dimethylethoxy)methylbenzene	95.8	95.8	0.0	^(b)^
*P. aeruginosa*	118	hydrogen cyanide 2-aminoacetophenone ammonia (dimer) 1-pentanol 1-undecene 2,4-dimethyl-1-heptene 1-decanol 2-propanol	64.9	92.9	87.3	91.9
*S. aureus*	42	ethyl 2-methylbutyrate 1,1,2,2-tetrachloroethane 1,4-pentadiene 1-hydroxy-2-propanone 2,3,4,5-tetrahydropyridazine 4-methylhexanoic acid butyl 2-methylbutanoate pyrimidine	87.5	90.5	26.1	91.7
*S. epidermidis*	5	(1,1-dimethylethoxy)methylbenzene	98.5	98.5	0.0	^(b)^
*S. pneumoniae*	10	3-phenylfuran	97.0	98.5	50.0	100.0

^(a)^By inspection of the publication where it was reported^19^, this compound is most likely a contaminant which was misidentified as bacterial VOC. ^(b)^Only the “not the pathogen” class examples was correctly classified, therefore the precision towards the “pathogen” class is not determinable.

In practice, the set of VOCs determined in the verification mode (Table 3) for each of the 11 microbial pathogens should be searched for in the sample’s headspace to predict the presence or absence of the pathogen. VOCs in each verification set can be exclusive from the pathogen, shared with others, or even not emitted by the pathogen. It is the combined information about the presence or absence of the VOCs in an unknown sample that is needed for the classifier to predict the presence of the pathogen in the sample, based on the training process. For example, to test a sample for the presence of *Mycobacterium tuberculosis*, information about the presence or absence of 4-methyldodecane, cymol and methyl nicotinate should be provided to this classifier. The classification result would be yes (“*Mycobacterium tuberculosis”*) or no (“not *Mycobacterium tuberculosis”* - any other of the 11 pathogens could be present), with no associated error (100% accuracy) (Table 3).

Due to the class unbalance, for pathogens with a reduced number of experiments the trivial classification as “not the pathogen” has already a high accuracy level. After running the classifier tests several times, there are, statistically, high chances of classifying it correctly in the “not the pathogen” class, and this results in a high average base (uninformed) accuracy (e.g., *Aspergillus niger, Clostridium difficile*).

The effect of training is reflected in the improvement of the classification accuracy (computed accuracy), for most pathogens (Table 3). The exceptions are *Proteus mirabilis* and *Staphylococcus epidermidis*, for which the model is not sensitive. In these cases, the high accuracy simply results from the statistical advantage of the model in classifying the examples as “not-*Proteus mirabilis*” or not-*Staphylococcus epidermidis*” This result suggests that the experiments associated with these pathogens do not carry enough information to enhance the separation beyond the obtained in an uninformed way. On the other hand, some pathogen classes are clearly separated from all others. Namely, *Pseudomonas aeruginosa*, *Mycobacterium tuberculosis* and *Haemophilus influenzae* (priority warning bacteria, as per the WHO^41^) are distinguished from all others with sensitivity and precision higher than 85% (Table 3), using only binary information about the respective verification VOCs sets.

Previous works also approached the concept of meta-search of microorganisms’ VOC signatures by accumulating data from several research studies on microbial VOCs^25,28,29,36^. However, besides having distinct scopes, the data processing methods employed were also distinct from those used in this work.

Bos *et al*.^25^ reviewed the VOCs produced by the six most relevant bacteria involved in sepsis and identified, through a pathogen-VOC association graph, pathogen-exclusive VOCs for three species (*Pseudomonas aeruginosa*, *Staphylococcus aureus* and *Escherichia coli*), which were proposed as biomarkers for those pathogens, in the context of sepsis.

The mVOC database, established in 2013^28^ and expanded in 2017 to mVOC 2.0^29^, summarizes microbial VOCs (human pathogens, plant pathogens, *fungi*, and soil-related microorganisms.) through “microorganism signature tables”, which are lists of VOCs emitted by the microorganisms. For an unknown sample’s VOC list, the putative emitter species can be inferred by manually searching and comparing microorganisms’ signatures. A significantly lower number of compounds is listed per microorganism than in the present work and the bibliographical sources are not easily assessible, which limits the understanding of the criteria for VOCs inclusion in the signatures listed for each microorganism.

The first report of machine learning application towards microorganism separation by VOCs using data published in the literature was from Abdullah *et al*.^36^. The authors developed a VOC database including plants, animals, *fungi* and bacteria. Then, they grouped microorganisms (bacteria and *fungi*) according to the similarity of the sets of emitted VOCs using unsupervised machine learning algorithms (clustering), which led to separation in pathogenic and non-pathogenic microorganisms for humans. This methodology does not allow to make predictions on the classification of new inputs because it is a grouping method.

The present work focused on VOC information associated with human pathogenic microorganisms, and supervised machine learning algorithms (SVM) were applied to perceive VOC patterns able to distinguish pathogen species, therefore allowing to make predictions over unknown input data.

Some of the VOCs retrieved by the machine-learning verification approach followed in the present work coincide with the putative biomarkers proposed by Bos and/or from the signature tables in mVOC 2.0 (Table S7). For example, the association *Escherichia coli* - indole and the association *Pseudomonas aeruginosa* – 1-undecene is common to the three studies. Hydrogen cyanide is related with *Pseudomonas aeruginosa* by Bos *et al*. and in this work. Methyl nicotinate is associated to *Mycobacterium tuberculosis*, and ethyl 2-methylbutyrate, 1-hydroxy-2-propanone, 2,3,4,5-tetrahydropyridazine and 4-methylhexanoic acid are associated to *Staphylococcus aureus* in this work and in the mVOC 2.0. Other compounds are structurally similar or belong to the same chemical classes, but are not identical between the three studies. For instance: 2-pentanone and 2-propanone are associated with *Escherichia coli* in this work and in mVOC 2.0, respectively.

The consistent finding of the similar pathogen-VOC associations in distinct databases using different data pre-processing and processing methods reinforces the discrimination power of these compounds.

It should be also noted that a recent review presented a compendium of 1840 VOC compounds identified from the breath and body fluids of healthy humans^5^, which should be considered if the aim is to diagnose the presence of a certain microbial pathogen directly from human samples without requiring cell cultivation.

### Refining pathogen classification in clinically relevant contexts

The 11 pathogens studied in this work are etiological agents of distinct infectious diseases, causing respiratory, urinary, skin, gastric or gastrointestinal infections (Table S8). According to the site of infection, the biological samples collected from patients and then tested for pathogen identification are distinct. For example, in respiratory infections it is common to analyse a sputum sample whereas when there is the suspicion of a urinary tract infection (UTI), urine is collected for microbiological analysis. Considering this, four case-studies were selected to illustrate possible refinements of the machine learning-driven pathogen classification (Table 4).

**Table 4:** Methodology refinement examples, using subgroups of the complete dataset. The classifier was run in verification and identification modes, using “leave-one-out” cross validation. The computed accuracy, sensitivity and precision represent performance measurements of the model.

Dataset name	Pathogen /Group of pathogens	Verification mode				Identification mode			
		VOCs set	Acc. (%)	Sens. (%)	Prec. (%)	VOCs	Av acc. (%)	Av sens. (%)	Av prec. (%)
Faeces	*Clostridium difficile* (6 experiments)	methyl 4-methylpentanoate 4-methylpentanoic acid 1-methyl-2-(1-methylethyl)-benzene	100	100	100	isopentanol 1-decanol hexanoic acid 2-methylbutanal 3-methylbutanoic acid 2-hexanone 4-methylpentanoic acid hexanal propanoic acid	92.4	92.8	95.1
	*Escherichia coli* (84 experiments)	isopentanol 1-decanol 3-methylbutanoic acid ammonia 2-methylbutanal 1,4-pentadiene 1-methyl-2-(1-methylethyl)-benzene 4-methylpentanoic acid tridecen-2-one	91.6	95.2	91.9				
	*Staphylococcus aureus* (48 experiments)	isopentanol 1-decanol ammonia 1,1,2,2-tetrachloroethane 1,4-pentadiene 2-methylpropanal 2-phenylethanol 2-undecanone tridecen-2-one	92.4	80.9	91.9				
*E. coli* groups	GI (28 experiments)	1-dodecanol 1-butanol	88.4	100	77.7	1-dodecanol 1-decanol methanol 1,3-butadiene 1-methyl-naphtalene 1-octanol indole	86.9	87.0	87.9
	UTI (22 experiments)	1-decanol 1-butanol methanol	91.3	77.2	94.4				
	Others (19 experiments)	1-octanol (dimer) methanol	82.6	57.8	73.3				
Clinical Samples and clinical isolates	*Escherichia coli* (25 experiments)	1-decanol	87.1	48.0	100.0	1-decanol trimethylamine methanethiol ɣ-butyrolactone 1,3,5-trimethylbenzene 1-propanol 3-methyl-4-(1-methylethenyl)cyclohexane dimethyl disulfide hydrogen cyanide	86.1	79.4	85.7
	*Heamophilus influenza* (6 experiments)	ɣ-butyrolactone	99.0	83.3	100.0				
	*Mycobacterium tuberculosis* (6 experiments)	1,3,5-trimethylbenzene 3-pentanol methyl nicotinate	100.0	100.0	100.0				
	*Proteus mirabilis* (11 experiments)	trimethylamine	98.0	81.8	100.0				
	*Pseudomonas aeruginosa* (41 experiments)	2-aminoacetophenone 2-ethyl-1-hexanol 1-butanol hydrogen cyanide 2,4-dimethyl-1-heptene 2-propanol, methyl thiocyanate	96.0	95.1	95.1				
	*Staphylococcus aureus* *(5 experiments)*	3-methyl-4-(1-methylethenyl)cyclohexane 1,4-pentadiene	98.0	60.0	100.0				
	*Streptococcus pneumoniae* (7 experiments)	2-butenal	98.0	71.4	100.0				
Breath and respiratory fluid clinical samples and clinical isolates	*Haemophilus influenza* (5 experiments)	ɣ-butyrolactone	100.0	100.0	100.0	1,3-butadiene 2,3-butanedione	100.0	100.0	100.0
	*Pseudomonas aeruginosa* (15 experiments)	2,3-butanedione	100.0	100.0	100.0				
	*Streptococcus pneumoniae* (5 experiments)	1,3-butadiene	100.0	100.0	100.0				

Acc: computed accuracy; Av acc: average computed accuracy; Sens: computed sensitivity; Av sens: average computed sensitivity; Prec: computed precision; Av prec: average computed precision.

In the first case, only experiments with microorganisms that are likely to be found in faeces were selected and used as input to the classifiers. A very accurate (92%) distinction between *Clostridium difficile*, *Escherichia coli* and *Staphylococcus aureus* was achieved, with sensitivity of 93% and precision of 95%. The sets of VOCs selected for each pathogen led to verification accuracies of 100% for *Clostridium difficile* and 92% for both *Escherichia coli* and *Staphylococcus aureus*. The classifier presented also high sensitivity (>81%) and precision (>91%) towards the three pathogens (Table 4).

The second case consisted in the distinction of *Escherichia coli* strains. Some *Escherichia coli* strains colonising the normal flora of the human gastrointestinal tract may also act as opportunistic pathogens causing infections in other body sites, namely urinary tract infections. These strains are, however, distinct from those non-coloniser strains that cause specifically gastrointestinal disease. Noteworthy, laboratory modified strains were not considered in this more refined study due to the low probability of finding them among patient samples. After training the classifier with the refined dataset, *E coli* experiments were successfully discriminated between GI (strains causing gastrointestinal infections), UTI (strains causing urinary tract infections) and others (strains that cause extra intestinal infections, such as blood, respiratory and skin infections, and infant diarrhoea) with average computed accuracy, sensitivity and precision of 86%, 87% and 88%, respectively in the identification mode. In the verification mode, accuracies between 80% and 90%, sensitivities between 58% and 100%, and precisions between 73% and 94% were achieved (Table 4). This means that in a hypothetical sample of faeces, for instance, the classifier would allow to predict if the sample contains an *Escherichia coli* strain causing GI infection or simply *Escherichia coli* strains belonging to the GI flora and, most probably, present in the sample. The rapid and correct strain identification channelled towards a specific biological sample would simplify diagnosis and allow prompt and adequate treatment reducing the improper use of antibiotics.

Considering that VOCs emitted by laboratory reference strains can vary from those emitted directly by patient samples or clinical isolates obtained from those, we next evaluated the effect of inputting only experiments with samples of clinical origin (patient samples and clinical isolates), independently of the type of infection. Although the sources of the samples are quite diverse (urine, breath, respiratory fluid, blood and skin), the identification accuracy, sensitivity and precision are very good (86%, 79% and 93%, respectively) for a set of 9 VOCs, 3 of them coinciding with those that separate the full dataset (Table 2): 1-decanol (the most discriminating VOC in both cases), ɣ-butyrolactone and 1,3,5-trimethylbenzene. In the verification mode, the selected sets of VOCs also vary but some individual VOCs coincide with those selected when the full dataset was input (Table 3): ɣ-butyrolactone for *Haemophilus influenzae*, methyl nicotinate for *Mycobacterium tuberculosis*, hydrogen cyanide, 2-aminoacetophenone, 2,4-dimethyl-1-heptene and 2-propanol for *Pseudomonas aeruginosa* and 1,4-pentadiene for *Staphylococcus aureus*. The consistency of some VOCs suggests they are important to discriminate between pathogens, regardless of the strain origin (clinical *vs*. reference). On the other hand, by limiting the machine learning input to clinical origin data, the sensitivity of the classifier varied. Namely, it decreased for *Escherichia coli* and *Haemophilus influenzae*, but increased notably for *Proteus mirabilis*, *Pseudomonas aeruginosa*, *Staphylococcus aureus* and *Streptococcus pneumoniae*.

A final narrowing down of the input data was performed by selecting only experiments that used respiratory fluid and breath samples (associated with respiratory infections). The input dataset became limited in size; but it illustrates the applicability of the machine-learning approach followed in this work. In this case, two VOCs were sufficient to separate *Haemophilus influenzae*, *Pseudomonas aeruginosa* and *Streptococcus pneumoniae* both in identification and verification modes with excellent selectivity and precision (Table 4); and again ɣ-butyrolactone appears associated with *Haemophilus influenzae*. None of the VOCs selected to discriminate the three pathogens in identification mode coincides with those selected for the full dataset or for the clinical samples dataset, suggesting that the SVM-based classification can be tuned to find sample type-specific VOCs and increase the performance towards pathogen discrimination.

## Discussion

Due to the diversity of human microbial pathogens studied, the heterogeneity of the testing conditions and of the analytical methods used for VOC identification in sample headspaces, a large amount of microbial VOC data is publicly available through peer-reviewed scientific research publications. However, the interpretation of pooled data from independent studies to distinguish pathogens is still an unmet need, as a combined dataset contains more information than the individual datasets independently and includes experimental variability, thus reflecting the current scientific knowledge.

Machine learning is as a powerful approach to deal with such large and wide dataset, and automatizes the metasearch for pathogen-discriminating sets of VOCs by determining relevant VOC patterns or profiles for pathogen classification.

Here, data collected from research articles published between 1977 and 2016 (inclusive), was integrated in an extensive database relating human microbial pathogens with VOCs detected in biological samples. By using the database as input of a SVM-based algorithm integrated with features selection process, the discriminability of 11 pathogen species based on the emitted 702 distinct VOCs was analysed. The dimensionality of the pathogen-VOC problem was reduced by obtaining the minimal sets of VOCs that contribute the most to discriminate pathogens. In this way, meaningful VOC-pathogen associations were determined from the pooled published data.

The SVM classifier was applied in multiclass (identification) and in dual-class (verification) modes and showed good performance to distinguish pathogens despite the VOCs sample type and origin, analytical method used to detect the VOCs, or sample’s experimental conditions.

In the identification mode, the results show that binary information (presence or absence) about a set of 18 VOCs in a sample is sufficient to predict the identity of a pathogen, in average, with 77% accuracy (74% sensitivity and 89% precision). In the verification mode, the classifier returned, for each of the 11 pathogens, a set VOCs which allows to distinguish the pathogen from all the others with >90% accuracy and variable sensitivity and precision.

Due to the uneven research efforts dedicated to the 11 pathogens, the number of studies published is unbalanced. This has noticeable effects on the classification performance, namely in the classification sensitivity, which is higher towards the most studied pathogens. For example, in the verification mode, the classifier is very sensitive and precise (>85%) in the separation of *Pseudomonas aeruginosa*, *Mycobacterium tuberculosis* and *Haemophilus influenzae* from all other pathogens, reinforcing the discriminating power of the VOC sets determined for these microbes.

Despite the known error associated with the classifier’s predictions, this work indicates that certain VOCs should be searched for in samples for pathogen identification purposes.

Experimental confirmation of the emission of VOCs for pathogens with low number of examples would provide additional data to train the classification algorithms and thus increase the sensitivity of the classifiers. Also, additional information on VOC concentration would be another path to improve the discrimination capabilities of the classifier.

Since only 6% of the available pathogen-VOC data was collected directly from clinical samples (body fluids and breath), the classifier and sets of VOCs are mainly useful for researchers working with microbial cultures. In the future, with the updates in the literature, the classifier can be trained with the new data to expand its usability to clinical samples and determine sets of VOCs more useful for pathogen classification directly from body fluids and breath. The applicability of the machine-learning approach to specific datasets was demonstrated, one of the test cases being the small subset of data corresponding to VOCs collected from clinical samples.

## Conclusion

Given the worldwide health burden of infectious diseases and antimicrobial resistance, the development of technologies for fast infection detection is an urgent need. Non-invasive diagnostic devices, exploring the volatolomics of human microbial pathogens (such as electronic noses and gas sensors), have the potential to contribute to this challenge. To develop such accurate and precise devices, it is important to fingerprint VOCs capable of identifying microbial pathogen species. The current knowledge (1977–2016) on VOC data related with human microbial pathogens is distributed among 71 scientific articles, involving 79 microbial pathogen species and 792 distinct VOCs. The present work established the first comprehensive pathogen-VOC database that compiles detailed information about VOCs collected from distinct biological samples. Furthermore, artificial intelligence tools based on supervised machine learning were here applied to this dataset, facilitating microbial classification, an important and innovative step towards fast infection detection. Automatic classifiers were implemented and applied to the subset of the pathogen-VOC database corresponding to the 11 most studied pathogens (from which 8 are considered a global world threat by the WHO). This led to the finding of small sets of VOCs that can be searched for in biological samples to predict the identity of the contaminating pathogen.

Scientific knowledge is dynamic, and the machine learning based methodology followed in this work supports future updates of the pathogen-VOC database. Data from further experimental studies will enrich the classifiers input data, and thus contribute to the selection of better pathogen-discriminating VOC sets. With the data available so far, the sets of VOCs found in this work provide good pathogen discrimination and are important compounds for the research of fast and non-invasive infection detection. Namely, these VOCs can be the targets needed to increase the selectivity of future gas sensors and electronic noses for infection diagnostics, through the development of biorecognition elements for bionic noses.

## Methods

### Bibliographic revision and data collection

Relevant scientific publications were retrieved from searches performed during 2016 in the online databases Pubmed, Web of Science and Google Scholar. Four sets of search terms were used: (A) “volatile organic compounds + health + pathogen + breath + disease”, (B) “exhaled volatile organic compounds”, (C) “volatile biomarkers + disease”, and (D) “volatile organic compounds + health + disease + detection + human”. Each round of search involved one database and one set of search terms. In total ~4000 articles were retrieved. From these, review articles were not considered and duplicates were removed (Figure S1). Articles were selected for examination if the title and/or abstract described the investigation of VOCs emitted by microbial pathogens, within a clinically pertinent context. Data in the selected articles was included in the study whenever it fulfilled the inclusion criteria. Namely, the article’s subject should identify the pathogen(s) instead of just mentioning a disease’s name; quantitative or qualitative information regarding individual VOCs per pathogen should be stated instead of patterns for chemical classes of VOCs; the bacterial culture conditions should be stated, if applicable; and the analytical method used to detect volatiles should be described.

An initial extensive Pathogen-VOC database was compiled relating each reported pathogen with the corresponding VOCs, the type of sample where the VOCs were detected (clinical sample or clinical isolate form blood, breath, skin, urine, faeces or respiratory fluid; or reference strain cultures), the VOCs concentrations (when available) and the analytical method used to detect them. When applicable, information regarding the bacterial strain, culture conditions and incubation times before VOC analysis were also added to the database.

To facilitate data processing, the initial extensive database was re-organized in a more computationally-readable table to which a new parameter was added: “experiment”. Some articles included VOC results from more than one experimental condition: for example, results obtained with distinct growth media, with different analytical methods, or even with multiple bacterial strains. To account for these situations, for the same article, each dataset obtained in a specific experimental condition was considered as a distinct experiment (Table S2). The reported VOCs were grouped according to their chemical class. Some compounds could be fitted in more than one class. In these situations, one of those chemical classes was randomly chosen. For instance, methyl thiocyanate contains both nitrogen and sulfur and it was classified as a sulfur containing compound. The relative abundance of each class was calculated by dividing the number of hits of VOCs from each class by the total number of hits concerning all the classes.

1 $${Re}lative\,abundance\,( \% )=\frac{Number\,of\,hit{s}_{class}}{Total\,number\,of\,hits}\times 100$$

### Input dataset for network visualization and machine learning algorithms

From the reorganized table, a pathogen-VOC matrix was built using an automatic conversion algorithm designed for that purpose to avoid operator errors. The matrix was organized per experiments in lines, and VOCs in columns. Each line has the following data: experiment number, paper where it was reported, analytical method used, present (1) and absent (0) VOCs, and pathogen name (Table S9). Since concentrations of the emitted VOCs were not available in most of the publications, only the boolean indication of the detection of the VOCs (presence/absence) was considered for this study. This is the pathogen-VOC dataset, represented in Fig. 1.

Different data filters were applied to this matrix to select specific sets of pathogens for analysis. Namely, only data concerning pathogens with more than 4 reported experiments were used for machine learning purposes.

### Network visualization

The open source Cytoscape (version 3.5) platform software^59,60^ was used to generate interaction graphs in a network-like representation that facilitates the interpretation of data and the observation of relationships between pathogens and VOCs in the pathogen-VOC dataset matrix.

The “group attributes” layout was used to organize the network according to the classification of nodes and visualize the variability and number of VOCs in the network. VOC nodes are grouped by chemical class while pathogen nodes are grouped by type of pathogen (Gram positive, Gram negative, fungi and protozoa).

The “circular yFiles” layout was used to identify unique pathogen-VOC relationships and visualize the respective number of hits. In this layout, nodes which are connected to two or more other nodes are represented in the boundary of a circle composed by the edges that connect those nodes. The nodes which are connected to only one node are represented outside the circle. In this layout, pathogen nodes and nodes of VOCs shared between many pathogens are represented in the boundary, while nodes of VOCs associated with only one pathogen are outside the circle.

### Machine Learning

The open source “scikit-learn” toolbox for machine learning in Python was used to implement a set of computing steps (Fig. 1 and Figure S3) to generate classifiers and estimate the classification performance. For a given pathogen class, the classifiers’ sensitivity and precision towards that class are given by Eqs 2 and 3, respectively:

2 $$Sensitivity\,=\frac{number\,of\,correct\,predictions\,in\,that\,class}{number\,of\,experiments\,in\,that\,class}\times 100\,({\rm{ \% }})$$

3 $${\Pr }ecision=\,\frac{number\,of\,correct\,predictions\,in\,that\,class}{number\,of\,predictions\,as\,that\,class\,}\times 100\,( \% )$$

Mean sensitivity and precision correspond to the averaged values over all pathogen classes.

The overall accuracy of the classifier is given by the mean accuracy (Eq. 4):

4 $$Mean\,accuracy=1-\frac{number\,of\,incorrectly\,predicted\,pathogens}{total\,number\,of\,experiments}$$

The first computational step consisted in the generation of the binary vector of features (VOCs) from the pathogen-VOC dataset. Data was split in training and test datasets for validation purposes. Then, a feature selection mechanism was executed to identify a good subset of features that generated low classification error. The classifier was trained in the process to search for the best subset of features. The process ended with a validation step where the final error was calculated. The classifier performance was assessed by computing the confusion matrix based on data not used in the training phase, implementing the leave-one-out cross validation method.

In the classification process, a supervised machine learning-based classifier was used to separate the pathogens based on binary VOC input data. To choose the most adequate classification method, several standard classifiers^61,62^ were tested: decision trees, naive Bayes classifier, k-nearest neighbour classifier and support vector machines (SVM). The classification accuracy was 30%, 63%, 65% and 77%, respectively. Since in the tests the results generated by the SVM outperformed the other three classifiers, SVM with linear kernels was selected as the method to execute the classification and the feature selection process

The SVM^37^ classification method operates a transformation on the data projecting it to a higher dimension space than the original data structure and applies an optimization technique to find an optimal separation plan in the new transformed space, such that separation margin between two classes is maximized. The base learning process of the SVM optimizes the margin distance by selecting a separation plan of a two-class problem. In the context of the pathogen-VOC dataset, this process was replicated to each pair of pathogens.

The classification task was performed in two modes^62^ – multiclass and dual class. The selection of the best VOCs subset to separate pathogens was executed by a sequential forward feature selection mechanism^63^ implemented in both modes of classification and depicted in Figure S4. It starts with an empty vector of features and adds one feature at a time, growing the vector until the classification error stops decreasing.

In the case of multiclass, the feature selection was executed for all the classes, returning a vector of the best VOCs for separating the pathogens between them.

In the dual class problem, the feature selection was executed for each “pathogen *vs*. the others” case, returning, for each pathogen, the set of features that better separates the pathogen from all the others.

All results are reported based on a cross-validation mechanism where a training dataset was used to find the best features and train the classifier, and a testing dataset was used to report the classification accuracy, sensitivity and precision, as well as the confusion matrix. The cross-validation method used was the leave-one-out^64^ which removes only one pathogen example from the training data set (one line of the matrix) and tests the classification in this sample, that has never been presented to the classifier. The results are the average values of running this process for each pathogen example.

## Electronic supplementary material

Supporting Information

Supporting Dataset

## References

[1] Yoo SM, Lee SY (2016). Optical Biosensors for the Detection of Pathogenic Microorganisms. Trends Biotechnol..

[2] Broza YY, Mochalski P, Ruzsanyi V, Amann A, Haick H (2015). Hybrid Volatolomics and Disease Detection. Angew. Chemie - Int. Ed..

[3] Nakhleh, M. K. *et al*. Diagnosis and Classification of 17 Diseases from 1404 Subjects *via* Pattern Analysis of Exhaled Molecules. *ACS Nano* acsnano.6b04930, 10.1021/acsnano.6b04930 (2016). 10.1021/acsnano.6b04930 PMC5269643 28000444

[4] Carey JR (2011). Rapid identification of bacteria with a disposable colorimetric sensing array. J. Am. Chem. Soc..

[5] Costello, B. *et al*. A review of the volatiles from the healthy human body. *J. Breath Res*. **8** (2014). 10.1088/1752-7155/8/1/014001 24421258

[6] Amann A (2014). The human volatilome: volatile organic compounds (VOCs) in exhaled breath, skin emanations, urine, feces and saliva. J. Breath Res..

[7] Wang C (2014). Exhaled volatile organic compounds as lung cancer biomarkers during one-lung ventilation. Sci. Rep..

[8] Wang C (2014). Blood volatile compounds as biomarkers for colorectal cancer. Cancer Biol. Ther..

[9] Schnabel RM (2015). Analysis of volatile organic compounds in exhaled breath to diagnose ventilator-associated pneumonia. Sci. Rep..

[10] Banday KM (2011). Use of urine volatile organic compounds to discriminate tuberculosis patients from healthy subjects. Anal Chem.

[11] Arasaradnam RP (2014). Differentiating coeliac disease from irritable bowel syndrome by urinary volatile organic compound analysis - A pilot study. PLoS One.

[12] Audrain B, Farag MA, Ryu CM, Ghigo JM (2015). Role of bacterial volatile compounds in bacterial biology. FEMS Microbiology Reviews.

[13] Schmidt R, Cordovez V, de Boer W, Raaijmakers J, Garbeva P (2015). Volatile affairs in microbial interactions. ISME J..

[14] Bonifacio LD (2010). Towards the photonic nose: a novel platform for molecule and bacteria identification. Adv. Mater..

[15] Moens M (2006). Fast identification of ten clinically important micro-organisms using an electronic nose. Lett. Appl. Microbiol..

[16] Boots AW (2014). Identification of microorganisms based on headspace analysis of volatile organic compounds by gas chromatography-mass spectrometry. J. Breath Res..

[17] Dolch, M. E. *et al*. Gram negative and positive bacteria differentiation in blood culture samples by headspace volatile compound analysis. *J. Biol. Res*. 1–8, 10.1186/s40709-016-0040-0 (2016). 10.1186/s40709-016-0040-0 PMC4788920 26973820

[18] Rees CA, Shen A, Hill JE (2016). Characterization of the Clostridium difficile volatile metabolome using comprehensive two-dimensional gas chromatography time-of-flight mass spectrometry. J. Chromatogr. B.

[19] Abd El Qader A (2015). Volatile organic compounds generated by cultures of bacteria and viruses associated with respiratory infections. Biomed. Chromatogr..

[20] Allardyce RA, Hill AL, Murdoch DR (2006). The rapid evaluation of bacterial growth and antibiotic susceptibility in blood cultures by selected ion flow tube mass spectrometry. Diagn. Microbiol. Infect. Dis..

[21] Phillips M (2007). Volatile biomarkers of pulmonary tuberculosis in the breath. Tuberculosis.

[22] Charlotte S (1997). Volatile metabolites from some gram negative bacteria. Chemosphere.

[23] Cox CD, Parker J (1979). Use of 2-aminoacetophenone production in identification of Pseudomonas aeruginosa. J. Clin. Microbiol..

[24] Hayward NJ, Jeavons TH, Nicholson AJ, Thornton AG (1977). Development of specific tests for rapid detection of Escherichia coli and all species of Proteus in urine. J. Clin. Microbiol..

[25] Bos LDJ, Sterk PJ, Schultz MJ (2013). Volatile Metabolites of Pathogens: A Systematic Review. PLoS Pathog..

[26] Sethi S, Nanda R, Chakraborty T (2013). Clinical application of volatile organic compound analysis for detecting infectious diseases. Clin. Microbiol. Rev..

[27] Sohrabi M, Zhang L, Zhang K, Ahmetagic A, Wei MQ (2014). Volatile Organic Compounds as Novel Markers for the Detection of Bacterial Infections. Clin. Microbiol..

[28] Lemfack MC, Nickel J, Dunkel M, Preissner R, Piechulla B (2014). mVOC: a database of microbial volatiles. Nucleic Acids Res..

[29] Lemfack, M. C. *et al*. mVOC 2.0: a database of microbial volatiles. *Nucleic Acids Res*. 10.1093/nar/gkx1016 (2017).

[30] Muto-Fujita A (2017). Data integration aids understanding of butterfly–host plant networks. Sci. Rep..

[31] Libbrecht MW, Noble WS (2015). Machine learning applications in genetics and genomics. Nat. Rev. Genet..

[32] Castelvecchi D (2016). Can we open the black box of AI?. Nature.

[33] Nakhleh MK (2017). Diagnosis and Classification of 17 Diseases from 1404 Subjects via Pattern Analysis of Exhaled Molecules. ACS Nano.

[34] Crofts TS, Gasparrini AJ, Dantas G (2017). Next-generation approaches to understand and combat the antibiotic resistome. Nat. Rev. Microbiol..

[35] Han BA, Drake JM (2016). Future directions in analytics for Infectious disease intelligence. EMBO Rep..

[36] Abdullah AA (2015). Development and Mining of a Volatile Organic CompoundDatabase. Biomed Res. Int..

[37] Zhang T (2001). An Introduction to Support Vector Machines and Other Kernel-Based Learning Methods. AI Mag..

[38] Hayward NJ, Jeavons TH (1977). Assessment of technique for rapid detection of Escherichia coli and Proteus species in urine by head-space gas-liquid chromatography. J. Clin. Microbiol..

[39] Jünger M (2012). Ion mobility spectrometry for microbial volatile organic compounds: A new identification tool for human pathogenic bacteria. Appl. Microbiol. Biotechnol..

[40] Zhu J, Bean HD, Kuo YM, Hill JE (2010). Fast detection of volatile organic compounds from bacterial cultures by secondary electrospray ionization-mass spectrometry. J. Clin. Microbiol..

[41] *WHO Global priority list of antibiotic-resistant bacteria to guide research, discovery, and development of new antibiotics*. *WHO* (World Health Organization 2017).

[42] Thorn RMS, Reynolds DM, Greenman J (2011). Multivariate analysis of bacterial volatile compound profiles for discrimination between selected species and strains *in vitro*. J. Microbiol. Methods.

[43] Shestivska V (2011). Quantification of methyl thiocyanate in the headspace of Pseudomonas aeruginosa cultures and in the breath of cystic fibrosis patients by selected ion flow tube mass spectrometry. Rapid Commun. Mass Spectrom..

[44] Španěl P (2016). Do linear logistic model analyses of volatile biomarkers in exhaled breath of cystic fibrosis patients reliably indicate *Pseudomonas aeruginosa*infection. J. Breath Res..

[45] Dryahina K, Sovova K, Nemec A, Spanel P (2016). Differentiation of pulmonary bacterial pathogens in cystic fibrosis by volatile metabolites emitted by their *in vitro* cultures: Pseudomonas aeruginosa, Staphylococcus aureus, Stenotrophomonas maltophilia and the Burkholderia cepacia complex. J. Breath Res..

[46] Scotter JM, Allardyce RA, Langford VS, Hill A, Murdoch DR (2006). The rapid evaluation of bacterial growth in blood cultures by selected ion flow tube-mass spectrometry (SIFT-MS) and comparison with the BacT/ALERT automated blood culture system. J. Microbiol. Methods.

[47] Syhre M, Manning L, Phuanukoonnon S, Harino P, Chambers ST (2009). The scent of Mycobacterium tuberculosis - Part II breath. Tuberculosis.

[48] Phillips M (2010). Breath biomarkers of active pulmonary tuberculosis. Tuberculosis.

[49] Mgode GF (2012). Mycobacterium tuberculosis volatiles for diagnosis of tuberculosis by Cricetomys rats. Tuberculosis.

[50] Syhre M, Chambers ST (2008). The scent of Mycobacterium tuberculosis. Tuberculosis.

[51] Cheepsattayakorn A, Cheepsattayakorn R (2015). Breath Tests in Diagnosis of Pulmonary Tuberculosis. Recent Pat. Biotechnol..

[52] Phillips M (2012). Point-of-care breath test for biomarkers of active pulmonary tuberculosis. Tuberculosis.

[53] Barker M (2006). Volatile organic compounds in the exhaled breath of young patients with cystic fibrosis. Eur. Respir. J..

[54] Filipiak W (2012). Characterization of volatile metabolites taken up by or released from Streptococcus pneumoniae and Haemophilus influenzae by using GC-MS. Microbiol. (United Kingdom).

[55] Dolch ME (2016). Gram-negative and -positive bacteria differentiation in blood culture samples by headspace volatile compound analysis. J. Biol. Res..

[56] Garner CE (2007). Volatile organic compounds from feces and their potential for diagnosis of gastrointestinal disease. Faseb J..

[57] Chadwick, D. & Goode, J. Novartis Foundation. *Acetaldehyde-related pathology: bridging the trans-disciplinary divide*. (John Wiley 2007).

[58] Yokoi A (2015). Relationship between acetaldehyde concentration in mouth air and tongue coating volume. J Appl Oral Sci.

[59] Shannon P (2003). Cytoscape: A software Environment for integrated models of biomolecular interaction networks. Genome Res..

[60] Smoot ME, Ono K, Ruscheinski J, Wang PL, Ideker T (2011). Cytoscape 2.8: New features for data integration and network visualization. Bioinformatics.

[61] Bishop, C. M. *Pattern recognition and machine learning*. (Springer 2006).

[62] Murphy, K. P. Machine Learning: A Probabilistic Perspective. *MIT Press* 25. 10.1007/978-3-642-21004-4_10 (2012).

[63] Mao KZ (2004). Orthogonal Forward Selection and Backward Elimination. IEEE Transactions on systems, man, and cybernetics-part B: cybernetics.

[64] Wong T-T (2015). Performance evaluation of classification algorithms by k-fold and leave-one-out cross validation. Pattern Recognit..

