# Author Correction: Machine learning for the meta-analyses of microbial pathogens’ volatile signatures

**DOI:** 10.1038/s41598-018-24111-w

**Published:** 2018-04-20

**Authors:** Susana I. C. J. Palma, Ana P. Traguedo, Ana R. Porteira, Maria J. Frias, Hugo Gamboa, Ana C. A. Roque

**Affiliations:** 1 UCIBIO, REQUIMTE, Departamento de Química, Faculdade de Ciências e Tecnologia, Universidade Nova de Lisboa, 2829-516 Caparica, Portugal; 2 LIBPhys-UNL, Departamento de Física, Faculdade de Ciências e Tecnologia, Universidade Nova de Lisboa, 2829-516 Caparica, Portugal

Correction to: *Scientific Reports* 10.1038/s41598-018-21544-1, published online 20 February 2018

This Article contains errors in Table 1. In the HTML and PDF versions of this Article, the chemical structure for 1,3,5-trimethylbenzene is incorrect. Additionally, in the PDF version, the chemical structure for 3-methylbutanoic acid has been omitted. The correct Table 1 appears below.

**Table 1:** List of VOCs that lead to the best classification results in the identification mode of the classifier, considering the 11 pathogen – 702 VOC dataset and using “leave-one-out” cross validation. The VOCs are listed in descendent order of importance for the performance of the classifier. Classification accuracy improves gradually by the sequential addition of the VOCs in the list to the vector of features that is used to classify the samples.

Number of VOCs used	VOC	Chemical structure	Added identification accuracy (%)	Cumulative identification accuracy (%)
1	1-decanol		49.7	49.7
2	3-methylbutanal		3.3	53.0
3	ethyl acetate		3.0	56.0
4	1,3,5-trimethylbenzene		1.7	57.7
5	3-methylbutanoic acid		1.8	59.5
6	indole		2.4	61.9
7	isopentanol		3.0	64.9
8	1-undecene		2.7	67.6
9	2-methylbutanal		2.0	69.6
10	ɣ-butyrolactone		1.5	71.1
11	4-methylphenol		0.9	72.0
12	furan		1.2	73.2
13	cymol		0.9	74.1
14	methyl nicotinate		0.9	75.0
15	cyclohexanone		0.6	75.6
16	4-methylpentanoic acid		0.2	75.8
17	n-butyl acetate		0.4	76.2
18	1-butanethiol		0.3	76.5

