# Supplementary material for: Machine learning for the meta-analyses of microbial pathogens’ volatile signatures
Source: Sci Rep. 2018 Feb 20;8:3360. doi: 10.1038/s41598-018-21544-1 (PMC5820279; doi:10.1038/s41598-018-21544-1)
Supplement: Supplementary file 1 — Supporting Information [file 41598_2018_21544_MOESM1_ESM.pdf]

Supplementary Information

Machine learning for the meta-analyses of microbial pathogens´ volatile signatures

Susana I. C.J. Palma[1], Ana P. Traguedo[1], Ana R. Porteira[1], Maria J. Frias[1], Hugo Gamboa[2], Ana C.A. Roque[1]*

[1]UCIBIO, REQUIMTE, Departamento de Química, Faculdade de Ciências e Tecnologia, Universidade Nova de Lisboa, 2829-516 Caparica, Portugal

[2] LIBPhys-UNL, Departamento de Física, Faculdade de Ciências e Tecnologia, Universidade Nova de Lisboa, 2892-516 Caparica, Portugal

* email: cecilia.roque@fct.unl.pt

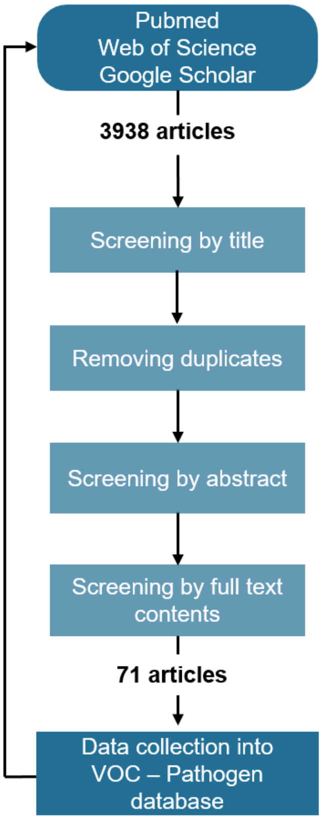

Figure S1. Procedure for selection of publications and relevant information to build a data Pathogen-VOC database.

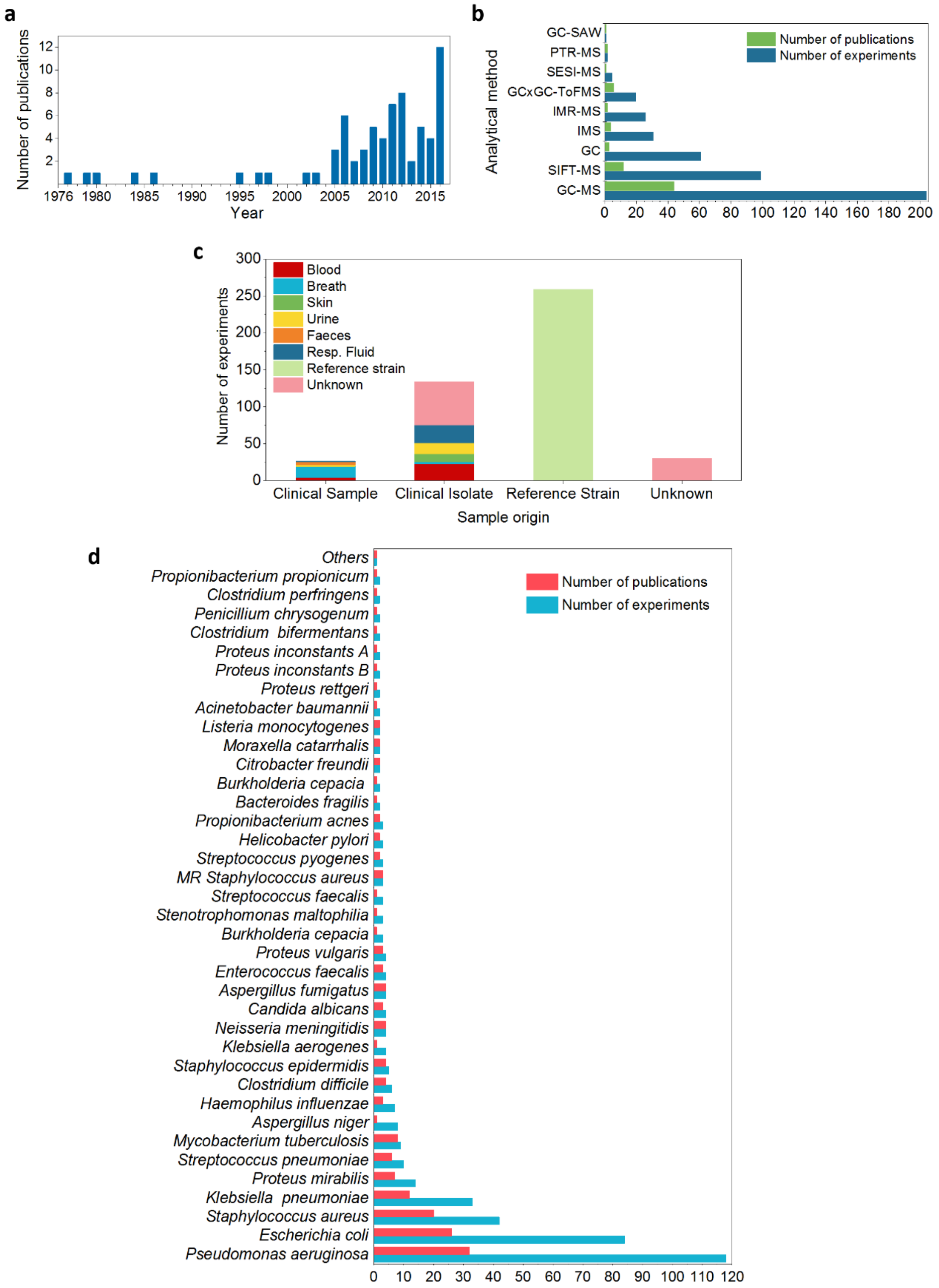

Figure S2. Description of the bibliography reviewed in this work. **(a)** Number of relevant scientific articles published per year regarding VOCs emitted by disease-associated microorganisms. **(b)** Analytical methods used to identify VOCs in the published experiments. **(c)** Origin of the biological samples used to identify VOCs. **(d)** Number of publications and experiments found per pathogen; "others" are pathogens

which were reported in only one publication, with only one experiment (*Staphylococcus lugdunensis, Streptococcus constellatus, Streptococcus gallolyticus, Actinomyces europaeus, Peptococcus niger, Actinomyces naeslundii, Clostridium ramosum, Clostridium sporogenes, Salmonella enterica typhi, Salmonella enterica paratyphi A, Staphylococcus capitis, Staphylococcus haemolyticus, Staphylococcus hominis, Klebsiella oxytoca, Enterococcus faecium, Enterobacter aerogenes, Streptococcus mitis, Salmonella enteritidis, Shigella flexneri, Salmonella enterica typhimurium, Acinetobacter baumannii, Plasmodium falciparum, Giardia duodenalis, Bacteroides capillosus, Bacteroides pyogenes Campylobacter jejuni, Clostridium septicum, Legionella pneumophila, Clostridium tertium. Eubacterium lentum, Eubacterium sp., Enterobacter cloacae, Fusobacterium simiae, Fusobacterium necrophorum, Nocardia sp., Morganella morganii, Peptostreptococcus anaerobius, Peptostreptococcus asaccharolyticus, Peptostreptococcus prevotii, Streptococcus agalactiae, Streptococcus viridans, Lactobacillus acidophilus.*

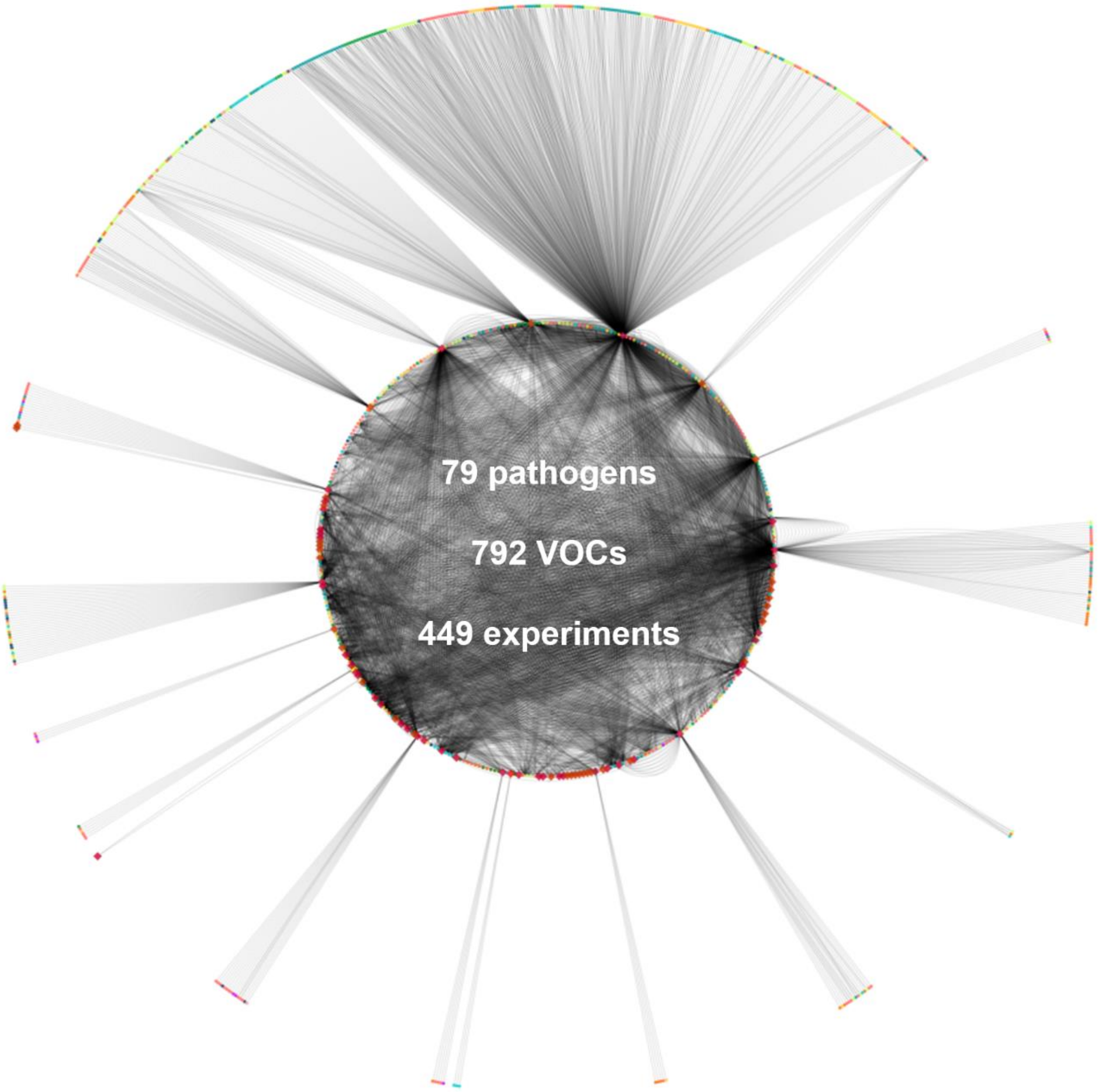

Figure S3. Graphical representation of pathogen-VOC associations described in the literature. In the graph, each line represents one hit for given a pathogen-VOC association. VOC nodes (dots) within the circumference represent VOCs that were reported to be emitted by multiple pathogens (shared VOCs), while VOC nodes outside the circle represent VOCs that were reported to be emitted by only one pathogen (exclusive VOCs). Pathogen nodes outside the circle represent pathogens for which only a single VOC has been reported.

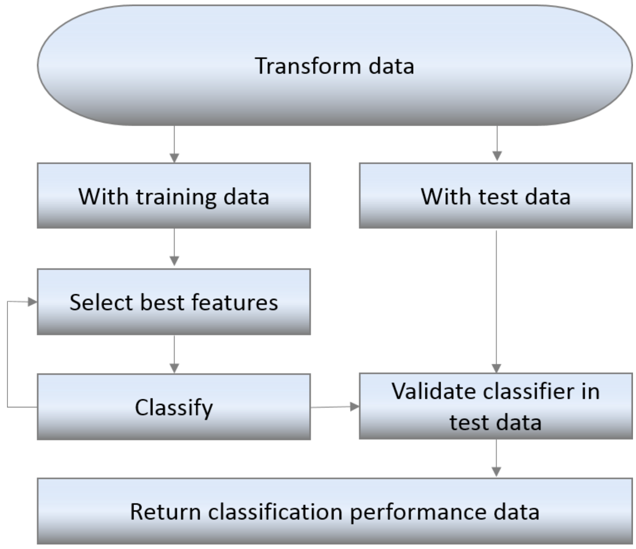

Figure S4. Classification steps.

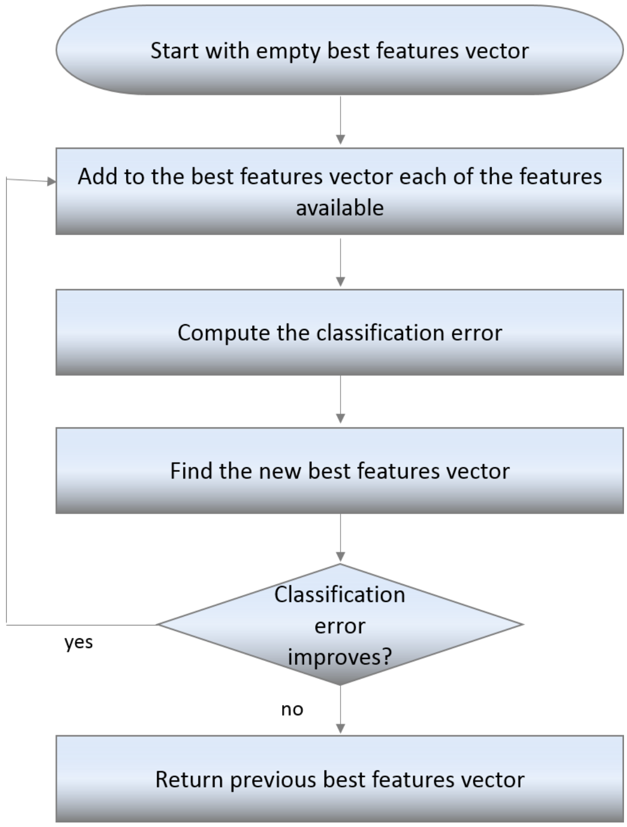

Figure S5. Features selection workflow.

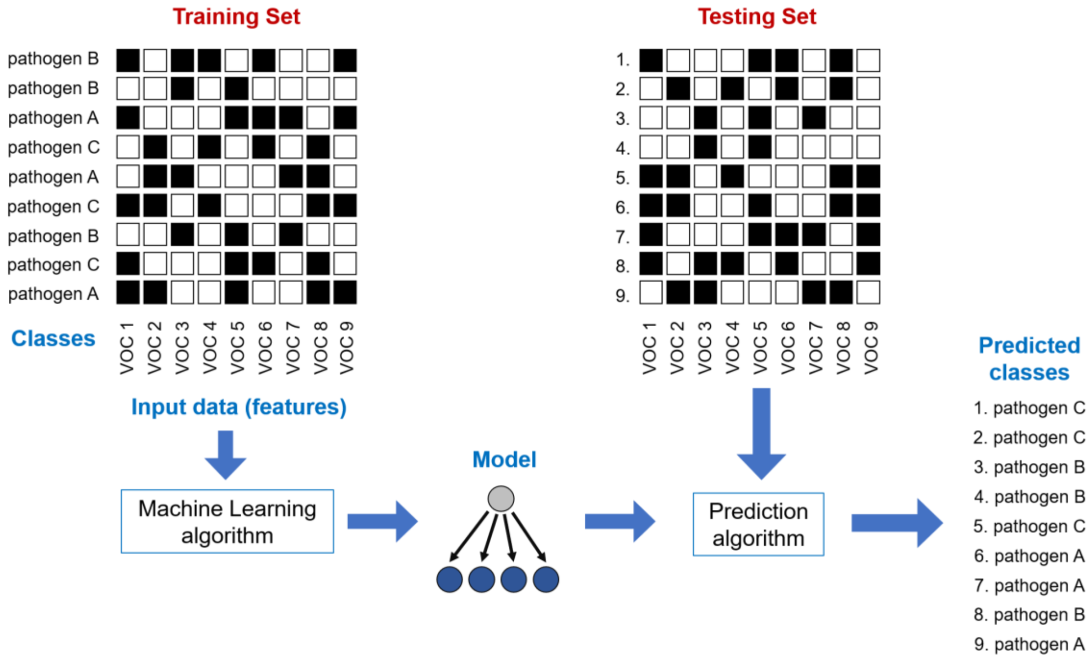

Figure S6. Concept of supervised machine learning in the context of this work. The dark and white squares represent the absence or presence of the various VOCs (column) in each example (line) (adapted from Libbrecht, M. W. *et al.*[1]).

Table S1. List of articles used as source for VOC-pathogen association data.

Article no.	Reference
1	Thorn, R., Reynolds, D. M. and Greenman, J. (2011) Multivariate analysis of bacterial volatile compound profiles for discrimination between selected species and strains in vitro.Journal of Microbiological Methods, 84 (2). pp. 258-264. ISSN 0167-7012 DOI: 10.1016/j.mimet.2010.12.001
2	Filipiak, Wojciech, et al. (2012)"Molecular analysis of volatile metabolites released specifically by Staphylococcus aureus and Pseudomonas aeruginosa." BMC microbiology DOI: 10.1186/1471-2180-12-113.
3	Boots AW, Smolinska A, van Berkel JJ, Fijten RR, Stobberingh EE, et al. (2014) Identification of microorganisms based on headspace analysis of volatile organic compounds by gas chromatography-mass spectrometry. J Breath Res 8: 027106. doi:10.1088/1752-7155/8/2/027106
4	J. Zhu, H.D. Bean, Y.M. Kuo, J.E. Hill, Fast detection of volatile organic compounds from bacterial cultures by secondary electrospray ionization-mass spectrometry (2010). J. Clin. Microbiol. 48, 4426–4431. doi:10.1128/JCM.00392-10.
5	M. Syhre, L. Manning, S. Phuanukoonnon, P. Harino, S.T. Chambers, The scent of Mycobacterium tuberculosis - Part II breath (2009), Tuberculosis. 89, 263–266. doi:10.1016/j.tube.2009.04.003.
6	Jünger, M., Vautz, W., Kuhns, M., Hofmann, L., Ulbricht, S., Baumbach, J. I., ... & Perl, T. (2012). Ion mobility spectrometry for microbial volatile organic compounds: a new identification tool for human pathogenic bacteria. Applied microbiology and biotechnology, 93(6), 2603-2614. doi: 10.1007/s00253-012-3924-4.
7	Kunze, N., Göpel, J., Kuhns, M., Jünger, M., Quintel, M., & Perl, T. (2013). Detection and validation of volatile metabolic patterns over different strains of two human pathogenic bacteria during their growth in a complex medium using multi-capillary column-ion mobility spectrometry (MCC-IMS). Applied microbiology and biotechnology, 97(8), 3665-3676. DOI 10.1007/s00253-013-4762-8.
8	Savelev, S. U., Perry, J. D., Bourke, S. J., Jary, H., Taylor, R., Fisher, A. J., ... & De Soyza, A. (2011). Volatile biomarkers of Pseudomonas aeruginosa in cystic fibrosis and noncystic fibrosis bronchiectasis. Letters in applied microbiology, 52(6), 610-613. DOI:10.1088/1752-7155/7/1/016003.
9	Barker, M., Hengst, M., Schmid, J., Buers, H. J., Mittermaier, B., Klemp, D., & Koppmann, R. (2006). Volatile organic compounds in the exhaled breath of young patients with cystic fibrosis. European respiratory journal, 27(5), 929-936. DOI: 10.1183/09031936.06.00085105.
10	Goeminne, P. C., Vandendriessche, T., Van Eldere, J., Nicolai, B. M., Hertog, M. L., & Dupont, L. J. (2012). Detection of Pseudomonas aeruginosa in sputum headspace through volatile organic compound analysis. Respir Res, 13, 87. doi: 10.1186/1465-9921-13-87.
11	Shestivska, V., Nemec, A., Dřevínek, P., Sovová, K., Dryahina, K., & Španěl, P. (2011). Quantification of methyl thiocyanate in the headspace of Pseudomonas aeruginosa cultures and in the breath of cystic fibrosis patients by selected ion flow tube mass spectrometry. Rapid Communications in Mass Spectrometry, 25(17), 2459-2467. doi: 10.1002/rcm.5146.
12	Storer, M. K., Hibbard-Melles, K., Davis, B., & Scotter, J. (2011). Detection of volatile compounds produced by microbial growth in urine by selected ion flow tube mass spectrometry (SIFT-MS). Journal of microbiological methods, 87(1), 111-113. doi: 10.1016/j.mimet.2011.06.012.
13	Tait, E., Perry, J. D., Stanforth, S. P., & Dean, J. R. (2014). Identification of volatile organic compounds produced by bacteria using HS-SPME-GC–MS. Journal of chromatographic science, 52(4), 363-373. doi:10.1093/chromsci/bmt042
14	Filipiak, W., Beer, R., Sponring, A., Filipiak, A., Ager, C., Schiefecker, A., ... & Amann, A. (2015). Breath analysis for in vivo detection of pathogens related to ventilator-associated pneumonia in intensive care patients: a prospective pilot study. Journal of breath research, 9(1), 016004. DOI: 10.1088/1752-7155/9/1/016004
15	W. Filipiak, A. Sponring, M. M. Baur, C. Ager, A. Filipiak, H. Wiesenhofer, M. Nagl, J. Troppmair, A. Amann (2012). Characterization of volatile metabolites taken up by or released from Streptococcus pneumoniae and Haemophilus influenzae by using GC-MS. Microbiology, 158, 3044. doi: 10.1099/mic.0.062687-0.
16	Abd El Qader, A., Lieberman, D., Shemer Avni, Y., Svobodin, N., Lazarovitch, T., Sagi, O., & Zeiri, Y. (2015). Volatile organic compounds generated by cultures of bacteria and viruses associated with respiratory infections.Biomedical Chromatography. DOI: 10.1002/bmc.3494.

Article no.	Reference
17	Chippendale, T. W., Gilchrist, F. J., Španěl, P., Alcock, A., Lenney, W., & Smith, D. (2014). Quantification by SIFT-MS of volatile compounds emitted by Aspergillus fumigatus cultures and in co-culture with Pseudomonas aeruginosa, Staphylococcus aureus and Streptococcus pneumoniae. Analytical Methods,6(20), 8154-8164. DOI: 10.1039/c4ay01217h
18	Perl, T., Jünger, M., Vautz, W., Nolte, J., Kuhns, M., Borg-von Zepelin, M., & Quintel, M. (2011). Detection of characteristic metabolites of Aspergillus fumigatus and Candida species using ion mobility spectrometry–metabolic profiling by volatile organic compounds. Mycoses, 54(6), e828-e837.doi: 10.1111/j.1439-0507.2011.02037.x
19	Tait, E., Perry, J. D., Stanforth, S. P., & Dean, J. R. (2014). Use of volatile compounds as a diagnostic tool for the detection of pathogenic bacteria. TrAc Trends in Analytical Chemistry, 53, 117-125.
20	Maddula, S., Blank, L. M., Schmid, A., & Baumbach, J. I. (2009). Detection of volatile metabolites of Escherichia coli by multi capillary column coupled ion mobility spectrometry. Analytical and bioanalytical chemistry, 394(3), 791-800. doi: 10.1007/s00216-009-2758-0.
21	Ulanowska, A., Kowalkowski, T., Hrynkiewicz, K., Jackowski, M., & Buszewski, B. (2011). Determination of volatile organic compounds in human breath for Helicobacter pylori detection by SPME-GC/MS. Biomedical Chromatography, 25(3), 391-397. DOI 10.1002/bmc.1460.
22	Ahmed, I., Greenwood, R., Costello Bde, L., Ratcliffe, N. M., & Probert, C. S. (2013). An investigation of fecal volatile organic metabolites in irritable bowel syndrome. PloS one, 8(3), e58204. doi:10.1371/journal.pone.0058204.
23	Garner, C. E., Smith, S., de Lacy Costello, B., White, P., Spencer, R., Probert, C. S., & Ratcliffe, N. M. (2007). Volatile organic compounds from feces and their potential for diagnosis of gastrointestinal disease. The FASEB Journal, 21(8), 1675-1688.
24	Cheepsattayakorn, A., & Cheepsattayakorn, R. (2014). Breath Tests in Diagnosis of Pulmonary Tuberculosis. Recent patents on biotechnology, 8(2), 172-175. DOI: 74/1872208309666140904115813
25	Phillips, M., Basa-Dalay, V., Bothamley, G., Cataneo, R. N., Lam, P. K., Natividad, M. P. R., ... & Wai, J. (2010). Breath biomarkers of active pulmonary tuberculosis. Tuberculosis, 90(2), 145-151. doi: 10.1016/j.tube.2010.01.003.
26	Phillips, M., Cataneo, R. N., Condos, R., Erickson, G. A. R., Greenberg, J., La Bombardi, V., ... & Tietje, O. (2007). Volatile biomarkers of pulmonary tuberculosis in the breath. Tuberculosis, 87(1), 44-52. doi: 10.1016/j.tube.2010.01.003.
27	Mgode, G. F., Weetjens, B. J., Nawrath, T., Lazar, D., Cox, C., Jubitana, M., ... & Kaufmann, S. H. (2012). Mycobacterium tuberculosis volatiles for diagnosis of tuberculosis by Cricetomys rats.Tuberculosis, 92(6), 535-542. doi: 10.1016/j.tube.2012.07.006
28	Phillips, M., Basa-Dalay, V., Blais, J., Bothamley, G., Chaturvedi, A., Modi, K. D., ... & Udwadia, Z. F. (2012). Point-of-care breath test for biomarkers of active pulmonary tuberculosis. Tuberculosis, 92(4), 314-320. doi: 10.1016/j.tube.2012.04.002.
29	Syhre, M., & Chambers, S. T. (2008). The scent of Mycobacterium tuberculosis. Tuberculosis, 88(4), 317-323. doi: 10.1016/j.tube.2008.01.002.
30	Banday, K. M., Pasikanti, K. K., Chan, E. C. Y., Singla, R., Rao, K. V. S., Chauhan, V. S., & Nanda, R. K. (2011). Use of urine volatile organic compounds to discriminate tuberculosis patients from healthy subjects. Analytical chemistry, 83(14), 5526-5534. doi: 10.1021/ac200265g.
31	Jia, B., Sohnlein, B., Mortelmans, K., Coggiola, M., & Oser, H. (2010). Distinguishing methicillin-resistant and sensitive Staphylococcus aureus using volatile headspace metabolites. Sensors Journal, IEEE, 10(1), 71-75. DOI: 10.1109/JSEN.2009.2035671
32	Bond, A., Vernon, A., Reade, S., Mayor, A., Wastling, J., Minetti, C., ... & Probert, C. (2015). PWE-173 Investigation of volatile organic compounds emitted from faeces for the diagnosis of giardiasis. Gut, 64(Suppl 1), A288-A288. DOI: http://dx.doi.org/10.15403/jgld.2014.1121.243.abo
33	Berna, A. Z., McCarthy, J. S., Wang, R. X., Saliba, K. J., Bravo, F. G., Cassells, J., ... & Trowell, S. C. (2015). Analysis of Breath Specimens for Biomarkers of Plasmodium falciparum Infection. The Journal of infectious diseases. doi: 10.1093/infdis/jiv176.
34	Neerincx, A. H., Geurts, B. P., Habets, M. F. J., Booij, J. A., van Loon, J., Jansen, J. J., ... & Wevers, R. A. (2016). Identification of Pseudomonas aeruginosa and Aspergillus fumigatus mono-and co-cultures based on volatile biomarker combinations. Journal of breath research, 10(1), 016002.
35	Allardyce, Randall A., et al. "Detection of volatile metabolites produced by bacterial growth in blood culture media by selected ion flow tube mass spectrometry (SIFT-MS)." Journal of microbiological methods 65.2 (2006): 361-365.

Article no.	Reference
36	Dolch, M. E., et al. "Volatile compound profiling for the identification of Gram-negative bacteria by ion-molecule reaction–mass spectrometry." Journal of applied microbiology 113.5 (2012): 1097-1105.
37	Preti, George, et al. "Volatile compounds characteristic of sinus-related bacteria and infected sinus mucus: analysis by solid-phase microextraction and gas chromatography–mass spectrometry." Journal of Chromatography B877.22 (2009): 2011-2018.
38	Schöller, Charlotte, Søren Molin, and Ken Wilkins. "Volatile metabolites from some gram-negative bacteria." Chemosphere 35.7 (1997): 1487-1495.
39	Bunge, Michael, et al. "On-line monitoring of microbial volatile metabolites by proton transfer reaction-mass spectrometry." Applied and environmental microbiology 74.7 (2008): 2179-2186.
40	Wood, William L., et al. "Analysis of volatile bacterial metabolites by gas chromatography-mass spectrometry." Spectroscopy 21.6 (2006).
41	Carroll, Will, et al. "Detection of volatile compounds emitted by Pseudomonas aeruginosa using selected ion flow tube mass spectrometry." Pediatric pulmonology 39.5 (2005): 452-456.
42	Cox, Charles D., and J. Parker. "Use of 2-aminoacetophenone production in identification of Pseudomonas aeruginosa." Journal of clinical microbiology 9.4 (1979): 479-484.
43	Labows, JOHN N., et al. "Headspace analysis of volatile metabolites of Pseudomonas aeruginosa and related species by gas chromatography-mass spectrometry." Journal of Clinical Microbiology 12.4 (1980): 521-526.
44	Hayward, N. J., et al. "Development of specific tests for rapid detection of Escherichia coli and all species of Proteus in urine." Journal of clinical microbiology 6.3 (1977): 195-201.
45	J. Gao, Y. Zou, Y. Wang, F. Wang, L. Lang, P. Wang, Y. Zhou, K. Ying, Breath analysis for noninvasively differentiating Acinetobacter baumannii ventilator-associated pneumonia from its respiratory tract colonization of ventilated patients.(2016). J. Breath Res. 10 , 27102. doi:10.1088/1752-7155/10/2/027102.
46	Costa, C. P., Gonçalves Silva, D., Rudnitskaya, A., Almeida, A. & Rocha, S. M. (2016). Shedding light on Aspergillus niger volatile exometabolome. Sci. Rep. 6:27441. DOI: 10.1038/srep27441
47	M.E. Dolch, S. Janitza, A.-L. Boulesteix, C. Graßmann-Lichtenauer, S. Praun, W. Denzer, G. Schelling, S. Schubert (2016). Gram-negative and -positive bacteria differentiation in blood culture samples by headspace volatile compound analysis, J. Biol. Res. 23 . doi:10.1186/s40709-016-0040-0.
48	C.A. Rees, A. Shen, J.E. Hill, Characterization of the Clostridium difficile volatile metabolome using comprehensive two-dimensional gas chromatography time-of-flight mass spectrometry (2016). J. Chromatogr. B. 1039, 8–16. doi:10.1016/j.jchromb.2016.11.009.
49	C.A. Rees, A. Smolinska, J.E. Hill, The volatile metabolome of Klebsiella pneumoniae in human blood, J. Breath Res. 10 (2016) 27101. doi:10.1088/1752-7155/10/2/027101.
50	P. Španěl, K. Sovová, K. Dryahina, T. Doušová, P. Dřevínek, D. Smith, Do linear logistic model analyses of volatile biomarkers in exhaled breath of cystic fibrosis patients reliably indicate Pseudomonas aeruginosa infection? (2016). J. Breath Res. 10, 36013. doi:10.1088/1752-7155/10/3/036013.
51	K. Dryahina, K. Sovova, A. Nemec, P. Spanel, Differentiation of pulmonary bacterial pathogens in cystic fibrosis by volatile metabolites emitted by their in vitro cultures: Pseudomonas aeruginosa, Staphylococcus aureus, Stenotrophomonas maltophilia and the Burkholderia cepacia complex (2016). J. Breath Res. 10, 37102. doi:10.1088/1752-7155/10/3/037102.
52	J. Chen, J. Tang, H. Shi, C. Tang, R. Zhang, Characteristics of volatile organic compounds produced from five pathogenic bacteria by headspace-solid phase micro-extraction/gas chromatography-mass spectrometry (2016). J. Basic Microbiol. 9999, 1–10. doi:10.1002/jobm.201600505
53	C.A. Rees, F.A. Franchina, K. V Nordick, P.J. Kim, J.E. Hill, Expanding the Klebsiella pneumoniae volatile metabolome using advanced analytical instrumentation for the detection of novel metabolites, J. Appl. Microbiol. (2016). doi:10.1111/jam.13372
54	H.D. Bean, C.A. Rees, J.E. Hill, Comparative analysis of the volatile metabolomes of Pseudomonas aeruginosa clinical isolates (2016). J. Breath Res. 10, 47102. doi:10.1088/1752-7155/10/4/047102.
55	H.D. Bean, J.-M.D. Dimandja, J.E. Hill, Bacterial volatile discovery using solid phase microextraction and comprehensive two-dimensional gas chromatography–time-of-flight mass spectrometry (2012). J. Chromatogr. B. vol.901, 41–46. doi:10.1016/j.jchromb.2012.05.038.
56	A.H. Neerincx, B.P. Geurts, J. van Loon, V. Tiemes, J.J. Jansen, F.J.M. Harren, L.A.J. Kluijtmans, P.J.F.M. Merkus, S.M. Cristescu, L.M.C. Buydens, R.A. Wevers, Detection of Staphylococcus aureus in cystic fibrosis patients using breath VOC profiles (2016). J. Breath Res. 10, 46014. doi:10.1088/1752-7155/10/4/046014.

Article no.	Reference
57	A.J. Scott-Thomas, M. Syhre, P.K. Pattemore, M. Epton, R. Laing, J. Pearson, S.T. Chambers, 2-Aminoacetophenone as a potential breath biomarker for Pseudomonas aeruginosa in the cystic fibrosis lung (2010). BMC Pulm. Med. 10, 56. doi:10.1186/1471-2466-10-56.
58	R.A. Allardyce, A.L. Hill, D.R. Murdoch, The rapid evaluation of bacterial growth and antibiotic susceptibility in blood cultures by selected ion flow tube mass spectrometry (2006). Diagn. Microbiol. Infect. Dis. 55, 255–261. doi:10.1016/j.diagmicrobio.2006.01.031.
59	T.J. Davies, VOLATILE PRODUCTS FROM ACETYLCHOLINE AS MARKERS IN THE Gas chromatogmphy-mass spectrometry (1984) . J. Chromatogr. Biomed. Appl. 307 , 11–21.
60	B. Enderby, D. Smith, W. Carroll, W. Lenney, Hydrogen cyanide as a biomarker for Pseudomonas aeruginosa in the breath of children with cystic fibrosis (2009). Pediatr. Pulmonol., 44 142–147. doi:10.1002/ppul.20963.
61	T. Hamilton-Kemp, M. Newman, R. Collins, H. Elgaali, K. Yu, D. Archbold, Production of the long-chain alcohols octanol, decanol, and dodecanol by Escherichia coli (2005). Curr. Microbiol. 51, 82–86. doi:10.1007/s00284-005-4469-x.
62	J. Julák, E. Procházková-Francisci, E. Stránská, V. Rosová, Evaluation of exudates by solid phase microextraction–gas chromatography (2003). J. Microbiol. Methods. 52, 115–122. doi:10.1016/S0167-7012(02)00148-3.
63	J. Kuzma, M. Nemecek-Marshall, W.H. Pollock, R. Fall, Bacteria produce the volatile hydrocarbon isoprene (1995). Curr. Microbiol. 30, 97–103. doi:10.1007/BF00294190.
64	J.M. Scotter, R.A. Allardyce, V.S. Langford, A. Hill, D.R. Murdoch, The rapid evaluation of bacterial growth in blood cultures by selected ion flow tube–mass spectrometry (SIFT-MS) and comparison with the BacT/ALERT automated blood culture system (2006). J. Microbiol. Methods. 65, 628–631. doi:10.1016/j.mimet.2005.09.016.
65	J.M. Zechman, S. Aldinger, J.N. Labows, Characterization of pathogenic bacteria by automated headspace concentration-gas chromatography (1986). J. Chromatogr. B Biomed. Sci. Appl. 377 49–57. doi:10.1016/S0378-4347(00)80760-4.
66	J. Julák, E. Stránská, V. Rosová, H. Geppert, P. Španěl, D. Smith, Bronchoalveolar lavage examined by solid phase microextraction, gas chromatography-mass spectrometry and selected ion flow tube mass spectrometry (2006). J. Microbiol. Methods. 65, 76–86. doi:10.1016/j.mimet.2005.06.009.
67	S.T. Chambers, M. Syhre, D.R. Murdoch, F. McCartin, M.J. Epton, Detection of 2-Pentylfuran in the breath of patients with Aspergillus fumigatus (2009). Med. Mycol. 47, 468–476. doi:10.1080/13693780802475212.
68	H. Elgaali, T.R. Hamilton-Kemp, M.C. Newman, R.W. Collins, K. Yu, D.D. Archbold, Comparison of long-chain alcohols and other volatile compounds emitted from food-borne and related Gram positive and Gram negative bacteria (2002). J. Basic Microbiol. 42, 373–380. doi:10.1002/1521-4028(200212)42:6<373::AID-JOBM373>3.0.CO;2-4.
69	J.W. Arnold, S.D. Senter, Use of digital aroma technology and SPME GC-MS to compare volatile compounds produced by bacteria isolated from processed poultry (1998) J. Sci. Food Agric. 78,343–348. doi:10.1002/(SICI)1097-0010(199811)78:3<343::AID-JSFA124>3.0.CO;2-5.
70	U. Siripatrawan, Rapid differentiation between E. coli and Salmonella Typhimurium using metal oxide sensors integrated with pattern recognition (2008), Sensors Actuators B Chem. 133, 414–419. doi:10.1016/j.snb.2008.02.046.
71	M. Lechner, A. Karlseder, D. Niederseer, P. Lirk, A. Neher, J. Rieder, H. Tilg, *H. pilory* infection increases levels of exhaled nitrate (2005). Helicobacter. 10, 385–390. doi:10.1111/j.1523-5378.2005.00345.x.

Table S2. Table of pathogen-VOC interactions collected from the 71 articles retrieved from the literature review, organized per experiments. **(available as a separate excel sheet).**

Table S3. Most referred VOCs (top hit VOCs) per pathogen and degree of exclusivity.

Pathogen	Top VOC	hits	exclusivity
Aspergillus niger	torreyol<br>α-methylionone<br>endobornyl acetate<br>6,10-dimethyl-,5,9-undecadien-2-one<br>(E)-3-nonen-2-one<br>2-methyl-6-phenyl-1,6-heptadiene<br>biphenyl<br>2-ethyl-1,4-dimethylbenzene	8	**exclusive**
Clostridium difficile	propanoic acid	4	shared with *E. coli, H. influenzae, K. pneumoniae, P. mirabilis, P. aeruginosa, S. epidermidis, S. aureus, S. penumoniae*
Mycobacterium tuberculosis	1-methyl-naphthalene	3	shared with *E. coli and M. tuberculosis*
	tridecane		shared with *Aspergillus niger*
	1,3,5-trimethylbenzene		
	**4-methyldodecane**		**exclusive**
	1-methyl-naphthalene		shared with *E. coli* and *M. tuberculosis*
	**cymol**		**exclusive**
	**methyl nicotinate**		**exclusive**
Klebsiella pneumoniae	isopentanol	22	shared with *A. niger*, *E. coli*, *H. influenzae*, *P. mirabilis*, *P.aeruginosa* and *S. aureus*
Streptococcus pneumoniae	acetaldehyde	9	**Shared with all others**
Haemophilus influenzae	acetaldehyde	5	
	butanal		shared with *C. difficile*, *P. aeruginosa*, *S. aureus* and *S. pneumoniae*
	2,3-butanedione		shared with *A. niger*, *E. coli*, *K. pneumoniae*, *P. aeruginosa*, *S. aureus* and *S.pneumoniae*
	methyl methacrylate		shared with *A. niger*, *P. aeruginosa*, *S. aureus* and *S. pneumoniae*
	methanethiol		shared with *S. pneumoniae*, *S. aureus*, *P. aeruginosa*, *P. mirabilis*, *K. pneumoniae*, *E. coli* and *C. difficile*
	dimethyl disulfide		shared with *S. pneumoniae*, *S. epidermidis*, *S. aureus*, *P. aeruginosa*, *P. mirabilis*, *E. coli*, *C. difficile* and *A. niger*
	carbon disulfide		shared with *S. pneumoniae*, *S. aureus*, *P. aeruginosa*, *K. pneumoniae* and *C. difficile*
	gama-butyrolactone		shared with *A. niger*
Proteus mirabilis	ethanol	10	shared with *S. pneumoniae*, *S. epidermidis*, *S. aureus*, *P. aeruginosa*, *K. pneumoniae* and *E. coli*
Escherichia coli	1-decanol	49	shared with *P. mirabilis, K. pneumoniae*, *C. difficile* and *A. niger*
Staphylococcus epidermidis	acetic acid	4	shared with *S. pneumoniae*, *S. aureus*, *P. aeruginosa, P. mirabilis*, *K. pneumoniae*, *H. influenzae*, *E. coli* and *C. difficile*
Pseudomonas aeruginosa	acetone	62	shared with *S. pneumoniae*, *S. epidermidis*, *S. aureus*, *P. mirabilis*, *K. pneumoniae*, *E. coli* and *A. niger*

Pathogen	Top VOC	hits	exclusivity
Staphylococcus aureus	isopentanol	22	shared with *A. niger*, *E. coli*, *H. influenzae*, *P. mirabilis* and *P. aeruginosa*

Table S4. Most referred exclusive VOCs per pathogen and respective number of hits.

Pathogen	Top exclusive VOCs	Number of hits	Refs.
Aspergillus niger	Torreyol	8	2
	α-methylionone	8	2
	endobornyl acetate	8	2
	6,10-dimethyl-,5,9-undecadien-2-one	8	2
	(E)-3-nonen-2-one	8	2
	2-methyl-6-phenyl-1,6-heptadiene	8	2
	biphenyl	8	2
	2-ethyl-1,4-dimethylbenzene	8	2
Staphylococcus aureus	4-methylhexanoic acid	1	3
	2,3,4,5-tetrahydropyridazine	1	4
	1,1,2,2-tetrachloroethane	1	5
	2-ethylacrolein	1	6
	1-hydroxy-2-propanone	1	6
	ethyl isovalerate	1	6
	1,4-pentadiene	1	7
	2-methylnaphthalene	1	7
	pyrimidine	1	8
	butyl 2-methylbutanoate	1	9
Pseudomonas aeruginosa	2-propanol	1	10
		35	11
Staphylococcus epidermidis	-	-	-
Escherichia coli	1-octanol (dimer)	13	11
Proteus mirabilis	-	-	-
Haemophilus influenzae	ethyl methyl sulfide	3	12
Streptococcus pneumoniae	3-phenylfuran	5	12
Klebsiella pneumoniae	3Z-octenyl acetate	2	12
	diethylether	2	13
	2-methylpropyl formate	2	13
	2,2,4,4-tetramethyloxolane	2	13
	2-methyl-2-hexanol	2	13
	4-penten-1-yl acetate	2	13
	4-methyl-1-propan-2-ylbicyclo[3.1.0]hexane (Thujane)	2	13
	2,6,11-trimethyldodecane	2	13
Mycobacterium tuberculosis	methyl nicotinate	3	14–16
	cymol	3	16–18
	4-methyldodecane	3	17,19,20
Clostridium difficile	methyl 4-methylpentanoate	2	21
		1	22

Table S5. VOCs that are shared between 9 or more pathogens of the filtered dataset. “x” in a given cell indicates that the VOC in the corresponding line was detected in samples of the pathogen in the corresponding column.

**VOC**	#hits	*A. niger*	*S. aureus*	*P. aeruginosa*	*S. epidermidis*	*E. coli*	*P. mirabilis*	*H. influenzae*	*S. pneumoniae*	*K. pneumoniae*	*M. tuberculosis*	*C. difficile*
dimethyl sulfide	72		x	x	x	x	x	x	x	x		x
dimethyl disulfide	70	*x*	x	x	x	x	x	x	x	x		x
acetic acid	54		x	x	x	x	x	x	x	x		x
acetaldehyde	48	*x*	x	x	x	x	x	x	x	x	x	x
1-propanol	31	*x*	x	x	x	x	x		x	x		x
toluene	29	*x*	x	x	x	x	x		x	x		x
ethyl acetate	25	*x*	x	x	x	x	x	x	x			x
methanol	23		x	x	x	x	x	x	x	x		x

Table S6. Performance of the SVM-based classifier in the identification (multiclass) mode, calculated for a classification using the 18 most discriminating VOCs and "leave-one-out" cross-validation. For a given pathogen class, the sensitivity of the classifier corresponds to the percentage of pathogens from that class that were identified; the precision corresponds to the percentage of predictions for that class that are correct.

**Pathogen class**	**Sensitivity (%)**	**Precision (%)**
Aspergillus niger	**100**	**100**
Clostridium difficile	**100**	**100**
Escherichia coli	**71**	**91**
Haemophilus influenzae	**71**	**100**
Klebsiella pneumoniae	**49**	**84**
Mycobacterium tuberculosis	**100**	**100**
Proteus mirabilis	**43**	**75**
Pseudomonas aeruginosa	**97**	**71**
Staphylococcus aureus	**69**	**62**
Staphylococcus epidermidis	**60**	**100**
Streptococcus pneumoniae	**50**	**100**
Average	**74**	**89**
Standard deviation	**22**	**14**

Table S7. Comparison between the sets of VOCs determined by the verification approach in this work, the mVOC 2.0 database and Bos *et al.* study. Consistently suggested VOCs are highlighted in green; VOCs structurally similar or belonging to the same chemical class are highlighted in blue.

**Pathogen**	**VOC set / fingerprints**		
	**This work**	**mVOC 2.0 database**[23,24]	**Bos *et al.* study**[25]
A. niger	(E)-3-nonen-2-one	1-hexanol<br>2-methylisoborneol<br>chalcogran<br>conophthorin<br>geosmin<br>3-Methylbutanoic acid i-pentylester<br>3-Methyl-2-butenoic acid ethylester<br>1-octen-3-ol<br>1-octene<br>2-octen-1-ol<br>2-pentanol<br>2-pentanone	
C. difficile	methyl 4-methylpentanoate<br>4-methylpentanoic acid<br>1-methyl-2-(1-methylethyl)-benzene	72 VOCs including:<br>methyl 4-methylpentanoate<br>4-methylpentanoic acid<br>1-ethyl-2-methylbenzene	
E.coli	indole<br>2-pentanone<br>1-octanol<br>1-ethyl-2-methylbenzene<br>isoamyl acetate<br>(Z)-7-tetradecen-1-ol<br>1-undecene<br>3-methylbutanoic acid<br>hexanal<br>phenylmethanol	47 VOCs including:<br>indole<br>2-pentanone<br>1-octanol<br>Isoamyl acetate<br>(Z)-7-tetradecen-1-ol<br>3-methylbutanoic acid	indole<br>methanol<br>1-pentanol<br>ethyl acetate
H. influenzae	ɣ-butyrolactone<br>1,2-bis(trimethylsilyl)benzene	20 VOCs including:<br>1,2-bis(trimethylsilyl)benzene	
K.pneumoniae	2,2,4,4-tetramethyloxolane<br>3Z-octenyl acetate<br>3-methylcyclohexene	56 VOCs	
M. tuberculosis	4-methyldodecane<br>cymol<br>methyl nicotinate	methyl phenylacetate<br>methyl 4-methoxybenzoate<br>methyl nicotinate<br>2-methoxy-1,1-biphenyl	
P.mirabilis	(1,1-dimethylethoxy)methylbenzene	2-methylpropan-1-amine<br>acetic acid	

P. aeruginosa	hydrogen cyanide<br>2-aminoacetophenone<br>ammonia (dimer)<br>1-pentanol<br>1-undecene<br>2,4-dimethyl-1-heptene<br>1-decanol<br>2-propanol	3-methyl-1-butanol<br>phenethyl alcohol<br>1-undecene<br>2-aminoacetophenone<br>dimethyldisulfide<br>dimethylsulfide<br>dimethylpyrazine<br>phenethyl alcohol<br>styrene	1-undecene<br>2,4-dimethylheptane<br>2-butanone<br>4-methyl-quinazoline<br>hydrogen cyanide<br>methyl thiocyanide
S. aureus	ethyl 2-methylbutyrate<br>1,1,2,2-tetrachloroethane<br>1,4-pentadiene<br>1-hydroxy-2-propanone<br>2,3,4,5-tetrahydropyridazine<br>4-methylhexanoic acid<br>pyrimidine	61 VOCs including:<br>3-methyl butanal<br>ethyl 2-methyl butanoate<br>1-hydroxy-2-propanone<br>3-methyl butyl acetate<br>2,3,4,5-tetrahydropyridazine<br>4-methylhexanoic acid	2-methylbutanal<br>3-methylbutanoic acid
S. epidermidis	(1,1-dimethylethoxy)methylbenzene	1-butanol<br>2-hydroxy-3-pentanone<br>2-methyl-1-butanol<br>2-methyl-butanal<br>2-methylbutanoic acid<br>2,3-butadione<br>3-hydroxy-2-butanone<br>3-methyl-1-butanol<br>3-methylbutanoic acid<br>acetic acid<br>isovaeric acid	
S. pneumoniae	3-phenylfuran	2-phenylethyl alcohol<br>acetic acid<br>benzaldehyde<br>benzyl alcohol<br>methyl mercaptan	

Table S8. Diseases/infections where the 11 studied pathogens are usually involved.

**Pathogen**	**Associated infections**														
	allergies	blood	bone	cystic fibrosis	gastric	gastrointestinal	heart	kidney	pneumonia	sinusitis	skin	tuberculosis	urinary tract	ear	respiratory tract
Aspergillus niger	x										x			x	x
Klebsiella pneumoniae		x	x						x				x		
Staphylococcus aureus		x	x	x	x		x		x	x	x		x		
Escherichia coli						x							x		
Streptococcus pneumoniae		x							x	x				x	
Haemophilus influenzae		x							x	x	x				
Mycobacterium tuberculosis												x			
Proteus mirabilis								x	x				x		
Pseudomonas aeruginosa		x	x	x		x	x		x	x			x		
Clostridium difficile						x									
Staphylococcus epidermidis							x						x		

Table S9. Example matrix demonstrating the structure of the Pathogen-VOC matrix used as input to the network visualization tool and machine learning algorithms.

**Experiment**	**Paper**	**Method**	**VOC 1**	**VOC 2**	**VOC 3**	⋯	**VOC 791**	**VOC 792**	**Pathogen**
1	1	SIFT − MS	1	0	1	…	0	1	*P. aeruginosa*
2	1	SIFT − MS	0	0	1	…	0	0	*S. aureus*
⋮	…	…	…	…	…	…	…	…	⋮
449	62	GC − MS	1	1	0	⋯	1	0	*E. coli*

References

1. Libbrecht, M. W. & Noble, W. S. Machine learning applications in genetics and genomics. *Nat. Rev. Genet.* **16,** 321–332 (2015).
2. Costa, C. P. *et al.* Shedding light on Aspergillus niger volatile exometabolome. *Sci. Rep.* **6,** 27441 (2016).
3. Preti, G. *et al.* Volatile compounds characteristic of sinus-related bacteria and infected sinus mucus: Analysis by solid-phase microextraction and gas chromatography-mass spectrometry. *J. Chromatogr. B Anal. Technol. Biomed. Life Sci.* **877,** 2011–2018 (2009).
4. Jia, B., Sohnlein, B., Mortelmans, K., Coggiola, M. & Oser, H. Distinguishing Methicillin-Resistant and Sensitive Staphylococcus aureus Using Volatile Headspace Metabolites. *IEEE Sens. J.* **10,** 71–75 (2010).
5. Boots, A. W. *et al.* Identification of microorganisms based on headspace analysis of volatile organic compounds by gas chromatography-mass spectrometry. *J. Breath Res.* **8,** 27106 (2014).
6. Filipiak, W. *et al.* Molecular analysis of volatile metabolites released specifically by Staphylococcus aureus and Pseudomonas aeruginosa. *BMC Microbiol.* **12,** 113 (2012).
7. Harren, F. Detection of Staphylococcus aureus in cystic fibrosis patients using breath VOC profiles. *J. Breath Res.* **10,** 46014 (2016).
8. Zhu, J., Bean, H. D., Kuo, Y. M. & Hill, J. E. Fast detection of volatile organic compounds from bacterial cultures by secondary electrospray ionization-mass spectrometry. *J. Clin. Microbiol.* **48,** 4426–4431 (2010).
9. Tait, E., Perry, J. D., Stanforth, S. P. & Dean, J. R. Identification of volatile organic compounds produced by bacteria using HS-SPME-GC-MS. *J. Chromatogr. Sci.* **52,** 363–373 (2014).
10. Barker, M. *et al.* Volatile organic compounds in the exhaled breath of young patients with cystic fibrosis. *Eur. Respir. J.* **27,** 929–936 (2006).
11. Shestivska, V. *et al.* Quantification of methyl thiocyanate in the headspace of Pseudomonas aeruginosa cultures and in the breath of cystic fibrosis patients by selected ion flow tube mass spectrometry. *Rapid Commun. Mass Spectrom.* **25,** 2459–2467 (2011).
12. Rees, C. A., Smolinska, A. & Hill, J. E. The volatile metabolome of *Klebsiella pneumoniae* in human blood. *J. Breath Res.* **10,** 27101 (2016).
13. Rees, C. A., Franchina, F. A., Nordick, K. V, Kim, P. J. & Hill, J. E. Expanding the Klebsiella pneumoniae volatile metabolome using advanced analytical instrumentation for the detection of novel metabolites. *J. Appl. Microbiol.* (2016). doi:10.1111/jam.13372
14. Mgode, G. F. *et al.* Mycobacterium tuberculosis volatiles for diagnosis of tuberculosis by Cricetomys rats. *Tuberculosis* **92,** 535–542 (2012).
15. Syhre, M. & Chambers, S. T. The scent of Mycobacterium tuberculosis. *Tuberculosis* **88,** 317–323 (2008).
16. Syhre, M., Manning, L., Phuanukoonnon, S., Harino, P. & Chambers, S. T. The

scent of Mycobacterium tuberculosis - Part II breath. *Tuberculosis* **89,** 263–266 (2009).

17. Phillips, M. *et al.* Breath biomarkers of active pulmonary tuberculosis. *Tuberculosis* **90,** 145–151 (2010).

18. Banday, K. M. *et al.* Use of urine volatile organic compounds to discriminate tuberculosis patients from healthy subjects. *Anal. Chem.* **83,** 5526–5534 (2011).

19. Cheepsattayakorn, A. & Cheepsattayakorn, R. Breath Tests in Diagnosis of Pulmonary Tuberculosis. *Recent Pat. Biotechnol.* **8,** 172–175 (2015).

20. Phillips, M. *et al.* Point-of-care breath test for biomarkers of active pulmonary tuberculosis. *Tuberculosis* **92,** 314–320 (2012).

21. Rees, C. A., Shen, A. & Hill, J. E. Characterization of the Clostridium difficile volatile metabolome using comprehensive two-dimensional gas chromatography time-of-flight mass spectrometry. *J. Chromatogr. B* **1039,** 8–16 (2016).

22. Garner, C. E. *et al.* Volatile organic compounds from feces and their potential for diagnosis of gastrointestinal disease. *Faseb J.* **21,** 1675–1688 (2007).

23. Lemfack, M. C., Nickel, J., Dunkel, M., Preissner, R. & Piechulla, B. mVOC: a database of microbial volatiles. *Nucleic Acids Res.* **42,** D744-8 (2014).

24. Lemfack, M. C. *et al.* mVOC 2.0: a database of microbial volatiles. *Nucleic Acids Res.* (2017). doi:10.1093/nar/gkx1016

25. Bos, L. D. J., Sterk, P. J. & Schultz, M. J. Volatile Metabolites of Pathogens: A Systematic Review. *PLoS Pathog.* **9,** 1–8 (2013).
